# The whole-genome dissection of root system architecture provides new insights for the genetic improvement of alfalfa (*Medicago sativa* L.)

**DOI:** 10.1093/hr/uhae271

**Published:** 2024-11-04

**Authors:** Xueqian Jiang, Xiangcui Zeng, Ming Xu, Mingna Li, Fan Zhang, Fei He, Tianhui Yang, Chuan Wang, Ting Gao, Ruicai Long, Qingchuan Yang, Junmei Kang

**Affiliations:** Institute of Animal Science, Chinese Academy of Agricultural Sciences, Beijing, China, 100193; Institute of Animal Science, Chinese Academy of Agricultural Sciences, Beijing, China, 100193; Institute of Animal Science, Chinese Academy of Agricultural Sciences, Beijing, China, 100193; College of Grassland Science, Qingdao Agricultural University, Qingdao, Shandong, China, 266109; Institute of Animal Science, Chinese Academy of Agricultural Sciences, Beijing, China, 100193; Institute of Animal Science, Chinese Academy of Agricultural Sciences, Beijing, China, 100193; Institute of Animal Science, Chinese Academy of Agricultural Sciences, Beijing, China, 100193; Institute of Animal Science, Ningxia Academy of Agricultural and Forestry Sciences, Yinchuan, Ningxia, China, 750000; Institute of Grassland Research, Chinese Academy of Agricultural Sciences, Hohhot, Inner Mongolia, China, 010000; Institute of Animal Science, Ningxia Academy of Agricultural and Forestry Sciences, Yinchuan, Ningxia, China, 750000; Institute of Animal Science, Ningxia Academy of Agricultural and Forestry Sciences, Yinchuan, Ningxia, China, 750000; Institute of Animal Science, Chinese Academy of Agricultural Sciences, Beijing, China, 100193; Institute of Animal Science, Chinese Academy of Agricultural Sciences, Beijing, China, 100193; Institute of Animal Science, Chinese Academy of Agricultural Sciences, Beijing, China, 100193

## Abstract

Appropriate root system architecture (RSA) can improve alfalfa yield, yet its genetic basis remains largely unexplored. This study evaluated six RSA traits in 171 alfalfa genotypes grown under controlled greenhouse conditions. We also analyzed five yield-related traits in normal and drought stress environments and found a significant correlation (0.50) between root dry weight (RDW) and alfalfa dry weight under normal conditions (N_DW). A genome-wide association study (GWAS) was performed using 1 303 374 single-nucleotide polymorphisms (SNPs) to explore the relationships between RSA traits. Sixty significant SNPs (−log_**10**_(*P*) ≥ 5) were identified, with genes within the 50 kb upstream and downstream ranges primarily enriched in GO terms related to root development, hormone synthesis, and signaling, as well as morphological development. Further analysis identified 19 high-confidence candidate genes, including AUXIN RESPONSE FACTORs (ARFs), LATERAL ORGAN BOUNDARIES-DOMAIN (LBD), and WUSCHEL-RELATED HOMEOBOX (WOX). We verified that the forage dry weight under both normal and drought conditions exhibited significant differences among materials with different numbers of favorable haplotypes. Alfalfa containing more favorable haplotypes exhibited higher forage yields, whereas favorable haplotypes were not subjected to human selection during alfalfa breeding. Genomic prediction (GP) utilized SNPs from GWAS and machine learning for each RSA trait, achieving prediction accuracies ranging from 0.70 for secondary root position (SRP) to 0.80 for root length (RL), indicating robust predictive capability across the assessed traits. These findings provide new insights into the genetic underpinnings of root development in alfalfa, potentially informing future breeding strategies aimed at improving yield.

## Introduction

The root system architecture (RSA) plays a crucial role in plant adaptation and productivity [1–3]. It is a complex network of physical structures, encompassing traits such as depth, branching, diameter, and spatial distribution [4, 5]. These morphological features have evolved to enable plants to efficiently absorb water and nutrients from diverse soil environments, thereby facilitating their adaptation to the local climate [5]. Importantly, RSA interacts with above-ground growth, influencing overall plant performance. Genotypes with well-developed root systems tend to achieve higher yields and biomass [6–8]. Furthermore, roots dynamically respond to changes in soil conditions on a cellular scale and in their architecture of the entire root system. For example, during water scarcity, the RSA undergoes morphological adjustments. Roots elongate and deepen, while branching angles decrease, enhancing their ability to access deep soil moisture and nutrients [9, 10]. Understanding the genetic basis of RSA and identifying root-related genes holds immense promise for the development of new crop varieties with improved agronomic traits.

The remarkable morphological plasticity of roots in the soil and the inherent challenge of directly measuring root traits due to their inaccessibility have resulted in a significant gap in our understanding of root biology and genetics [5, 11]. The well-established *Arabidopsis* model plant provides valuable insights into the fundamental mechanisms governing root growth, including meristem organization, lateral root development, gravitropism, and root responses to environmental factors [5]. Plant hormones play a coordinated role in regulating root development, with many of these hormones converging on the auxin signaling pathway, a pivotal guidance system for root growth [5, 12–14]. Among the key players are *AUXIN RESPONSE FACTORs* (*ARFs*), which act as transcriptional activators of early auxin-responsive genes. In the founder cells of primary roots, the interaction between *WUSCHEL-RELATED HOMEOBOX9* (*WOX9*) and *ARF5* initiates primary root development by activating RGIs in *Arabidopsis* [15]. Furthermore, *ARF7* and *ARF19* directly regulate lateral root formation by activating two *LATERAL ORGAN BOUNDARIES-DOMAIN* genes (*LBD16* and *LBD29*) [15, 16]. In recent years, breeders have sought to enhance crop performance through genetic modification of root architecture. In particular, genes such as *ZmRSA3.1* and *ZmRSA3.2*, associated with RSA, and *Enhanced Gravitropism 2* (*EGT2*), have been cloned and demonstrated to modulate root growth angles in maize and wheat, respectively [17, 18]. These advancements hold promise for increasing crop yield by improving water and nitrogen utilization efficiency while minimizing root-to-root competition.

Most studies have primarily focused on the morphological development of alfalfa (*Medicago sativa* L.) roots [11, 19, 20]. Unlike annual crops such as *Arabidopsis*, alfalfa is a perennial leguminous plant that undergoes multiple harvests yearly [21]. Consequently, understanding root growth patterns is crucial for the regrowth capacity and long-term persistence of alfalfa [19]. The root system of alfalfa can be classified into two main types: the taproot type, which has a dominant taproot and few lateral roots, and the branched root type, which has a less conspicuous taproot and numerous lateral roots [11]. Alfalfa plants typically possess deep taproots, capable of reaching lengths of 6 m or more [22]. Furthermore, robust and lateral fibrous roots play vital roles in the overall structure of the alfalfa root system [19, 23]. These combined characteristics contribute to the complexity of alfalfa root structure and genetic factors.

Recent studies have highlighted the potential benefits of modifying the RSA of plants to enhance their stress tolerance and overall yields [3, 8, 24]. By doing so, penalties from trade-offs between growth and defense can be reduced or avoided, thereby improving yield and increasing stress tolerance. Therefore, it is crucial to understand how specific root traits are related to herbage yield and other adaptability traits. Existing literature on alfalfa has revealed varying correlations between root traits and yield, influenced by experimental conditions and genetic diversity [23, 25]. In particular, greenhouse trials involving progenies from high root weight selections demonstrated that increased root diameter, branching, and herbage yield were achievable [26]. Johnson *et al*. [23] delved into the relationship between alfalfa RSA traits and forage yield. Their findings indicated moderate to high heritability for the measured root traits, with taproot diameter emerging as a strong predictor of forage yield (correlation coefficient up to 0.75). Additionally, two cycles of divergent selection led to the creation of populations with distinct RSA: taprooted and branch-rooted. Interestingly, populations with greater root mass showed positive correlations with forage yield, whereas their deep taproots enabled better access to water resources, thus enhancing drought tolerance [27]. Meanwhile, cold-hardy alfalfa varieties, compared to non-cold-hardy varieties, tend to exhibit thicker diameters of both taproot and lateral roots, and a larger number of lateral roots [28]. In addition to traditional breeding approaches, molecular interventions have shown promise. Overexpression of *Glycine soja WRKY20* and *ZFP1* in transgenic alfalfa increased tolerance to drought by promoting root length [29, 30]. Similarly, miR156-overexpressing plants exhibit enhanced above-ground biomass and well-developed root systems, resulting in improved drought resistance and overall biomass compared to wild-type plants under stress conditions [31, 32]. In summary, these studies suggest that manipulating alfalfa root growth and development is a feasible and effective way to enhance both yield and abiotic stress tolerance.

The roots of alfalfa are buried underground, making them difficult to select during breeding. However, the root system is also indirectly affected by the selection of alfalfa based on the above-ground phenotype, due to its close relationship with the above-ground part [27, 28]. Traditional breeding based on phenotypic selection faces the challenge of selecting breeding materials with desirable RSA types to meet production needs, as it involves high uncertainty and low efficiency. Marker-assisted selection (MAS) breeding is an effective method for overcoming these issues [33]. Therefore, elucidating the genetic basis of root structure in alfalfa will facilitate breeding new alfalfa varieties with suitable root structures through MAS. Genome-wide association studies (GWAS) have been utilized for the genetic analysis of complex quantitative traits [34, 35]. In addition, genomic prediction (GP) can enhance selection accuracy and efficiency, and thus increase genetic gain compared to phenotypic selection [36]. Recent studies have demonstrated the feasibility and improvement of using GWAS-derived markers for GP [37–40]. GWAS and GP have advanced the investigation of key traits related to yield and quality in alfalfa [37, 41–43]. Various machine learning techniques, such as support vector machines, random forests, and deep learning, have been applied to GP, which can enhance the GP methodology. A machine-learning-based GP of alfalfa fall dormancy was conducted using GWAS-associated markers. The highest prediction accuracy (PA, 0.64) was achieved by combining the top 3000 GWAS markers with the support vector machine (SVM) regression method [37].

Previous research on RSA in crops has highlighted the importance of genetic factors in root development [17, 18]. Yet, there remains a significant gap in our understanding of these genetic influences in alfalfa. This study aimed to fill this gap by exploring the genetic basis of RSA in alfalfa, offering new perspectives for crop improvement. This study evaluated six RSA traits in an alfalfa natural-variation population comprising 171 germplasm materials in a greenhouse. This study aimed to: (i) determine the relationship between alfalfa RSA traits and their yield and drought resistance; (ii) identify single nucleotide polymorphisms (SNPs) and candidate genes associated with RSA traits through GWAS and evaluate the potential of these SNPs' favorable haplotypes for alfalfa breeding and (iii) use the GP method of machine learning to predict RSA traits. Our results offer valuable insights into the regulation of RSA and other important agricultural traits in alfalfa and other legume crops.

## Results

### RSA traits differ among association panel

Root system samples (after the removal of fibrous roots) were used to measure root phenotypes, and six root traits were measured for each sample: RN, TRD, SRD, RDW, RL, and SRP (Table S1). The mean values of the six clonal plants for each trait were used to estimate the root phenotypes of each genotype. The six RSA traits showed a nearly normal distribution (Fig. S1 A). The broad-sense heritability (H^2^) was 53.79% for SRP, which was lower than the heritability of the remaining five traits, with heritabilities ranging from 84.20% (RL) to 91.03% (TRD) (Table S3). These results demonstrated that alfalfa root development was primarily regulated by its genes in a relatively stable greenhouse environment.

**Figure 1:**
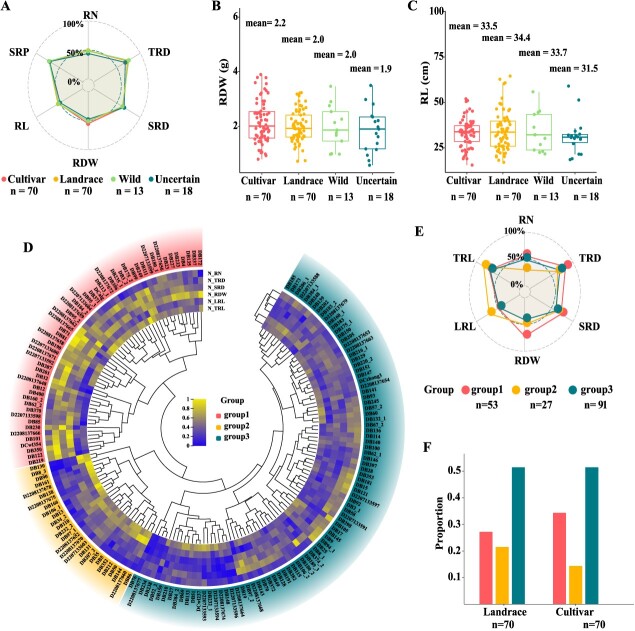
Phenotype analysis of 171 alfalfa samples based on six root system architecture (RSA) traits. (A) Comparison of six root traits among different improved statuses. (B, C) Box plots that display RDW and RL among different improved statuses, respectively. The mean values of the materials in different improved statuses are displayed. (D) Cluster analysis of 171 alfalfa samples based on six RSA traits. (E) Comparison of six root traits among the three clusters (groups 1–3). (F) Proportion of materials from three clusters (groups 1–3) in the landrace and cultivar. RN, root number; TRD, taproot diameter; SRD, secondary root diameter; RDW, root dry weight; RL, root length; SRP, secondary root position. Here, n represents the number of alfalfa materials in different categories.

Based on the improved status of the materials, we divided the association panel into four groups (wild, landrace, cultivar, and uncertain) (Table S1). Our phenotypic data showed that the six RSA traits showed large variations in the association panel, with coefficients of variation ranging from 15.89% (SRD) to 37.08% (RN) (Table S3). However, there were no significant differences in the six root traits among the different breeding materials (Fig. 1 A ~ C). We further applied PCA and cluster analysis to investigate the association between the six root traits. Out of the six principal components (PCs), the first two PCs explained 63.5% of the phenotypic variation (Fig. S1B). PC1 separated the two root length traits (RL and SRP) from the other four traits, consistent with the cluster analysis results (Fig. 1 D and Fig. S1C). In addition, the association panel was divided into three groups according to the cluster analysis of 171 alfalfa samples based on six RSA traits (Fig. 1D and Table S1). We then compared the RSA traits among the three groups in the association panel; group1 showed the highest TRD, SRD, and heaviest RDW, while group2 had the smallest RN and longest RL (Fig. 1E). Group3 did not show any outstanding features compared with the other two groups. Further analysis found that from the local landrace to cultivar, the material proportion of group1 increased while the proportion of group2 decreased (Fig. 1 F). Consistent with this result, although there were no significant differences in RDW and RL between landraces and cultivars, cultivars showed greater RDW and shorter RL (Fig. 1B and C). This result suggest that people select larger and heavier root systems rather than longer ones in alfalfa breeding programs.

### Relationship between RSA traits and yield under normal and drought conditions

#### Relationship between the six RSA traits

**Figure 2:**
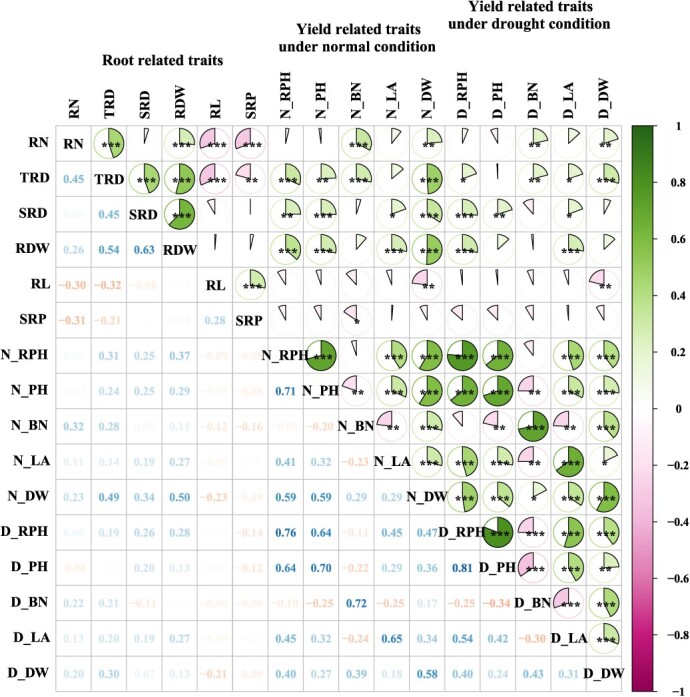
Correlations between root system architecture (RSA) traits and forage yield-related traits. The size of the pie chart indicates the strength of the correlation. Asterisks denote significance (^*^P < 0.05, ^**^P < 0.01, ^***^P < 0.001). RN, root number; TRD, taproot diameter; SRD, secondary root diameter; RDW, root dry weight; RL, root length; SRP, secondary root position; RPH, regeneration plant height; PH, plant height; BN, branch number; LA, leaf area; DW, dry weight. “N_” and “D_” symbolize normal and drought conditions, respectively. For example, N_DW and D_DW represent the forage dry weight under normal and drought conditions.

In the previous cluster and PCA analysis, we distinguished the two phenotypes related to root length from the remaining four phenotypes. Correlation analysis of the six root traits confirmed these results (Fig. 2). There was a positive correlation between the two length-related traits, RL and SRP, with a correlation coefficient of 0.28 (*P* < 0.001). They had a negative correlation with RN and TRD (−0.21 ~ −0.32), and had no correlation with SRD and RDW. Low to moderate correlations were observed in the remaining four RSA traits (no significant correlation between RN and SRD), with correlation coefficients ranging from 0.26 to 0.63. RN, TRD, and SRD collectively influenced RDW. Among them, the correlation between RN and RDW was the lowest at 0.26 (*P* < 0.001); the correlation between TRD and RDW was 0.54 (*P* < 0.001); and the highest correlation was found between SRD and RDW at 0.63 (*P* < 0.001) (Fig. 2).

#### Relationship between RSA traits and yield under normal conditions

Roots play a key role in plant growth. As expected, we identified correlations between RSA traits and five yield-related traits under normal conditions (Fig. 2). Both N_RPH and N_PH were significantly correlated with TRD, SRD, and RDW, with correlations ranging from 0.24 to 0.37. N_BN was positively correlated with RN and TRD, and the correlation coefficients were 0.32 (*P* < 0.001) and 0.28 (*P* < 0.001), respectively, indicating that materials with more lateral roots and larger taproots have more branches. Dry weight (N_DW) was positively correlated with the four root traits of RN (0.23, *P* < 0.01), TRD (0.49, *P* < 0.001), SRD (0.34, *P* < 0.001), and RDW (0.50, *P* < 0.001), and negatively correlated with RL (−0.23, *P* < 0.01). The low to moderate correlation between root traits and above-ground traits directly selected by humans may explain why people choose larger, heavier roots over longer roots in alfalfa breeding.

#### Relationship between RSA traits and yield under drought stress

Root- and yield-related traits under drought conditions showed similar results (Fig. 2). However, we obtained smaller correlation coefficients between these phenotypes. It is worth noting that dry weight under drought conditions (D_DW) was not significantly correlated with SRD and RDW, whereas these two RSA traits were significantly correlated with N_DW. D_DW was positively correlated with RN and TRD, with correlation coefficients of 0.20 (*P* < 0.01) and 0.30 (*P* < 0.001), respectively, and negatively correlated with RL, with a correlation coefficient of −0.21 (*P* < 0.01).

Root structures with more RNs, larger taproots and lateral root diameters, heavier root biomass, and shorter root lengths tended to gain higher yield. Owing to the correlation between RDW, TRD, and dry weight, we selected these four phenotypes (RDW, TRD, N_DW, and D_DW) for cluster analysis and screened out 12 individuals with larger root systems. Moreover, they had higher yields under both normal and drought stress conditions (Fig. S2 and Table S1). These materials with developed root systems can be used to breed new alfalfa varieties with higher yields under both normal and drought conditions.

### GWAS for six RSA traits and identification of RSA candidate genes

**Figure 3:**
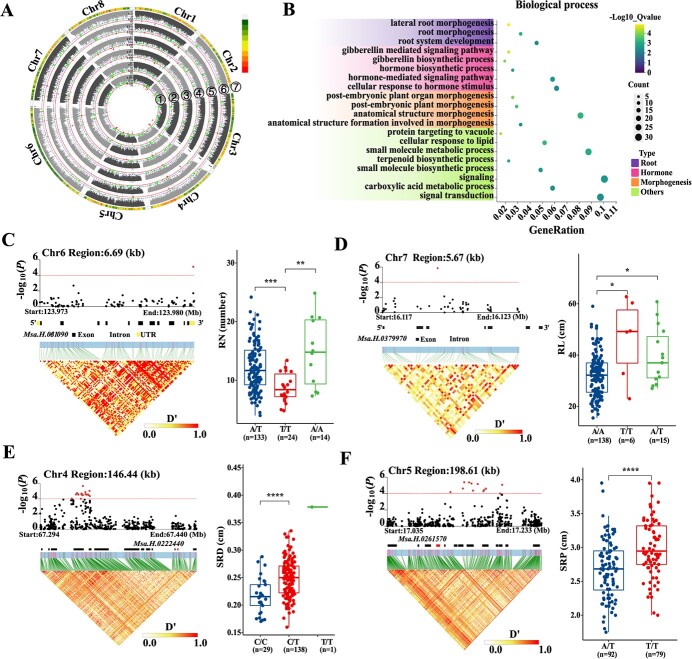
GWAS identification of candidate genes for variation in alfalfa root traits. (A) Circular Manhattan plots of the association analysis for the six root system architecture (RSA) traits using Blink. The dotted red line indicates the significance threshold of a *P-*value of 1 × 10^−5^. ①–⑥ represent different traits: ① RN, root number; ② TRD, taproot diameter; ③ SRD, secondary root diameter; ④ RDW, root dry weight; ⑤ RL, root length; and ⑥ SRP, secondary root position. ⑦ distribution of SNP markers on eight chromosomes in the association pool. The color represents the density of the SNP markers. (B) GO enrichment analysis of 308 GWAS candidate genes. These candidate genes were identified in our GWAS results within 50 kb of the significant SNPs. (C and D) GWAS identification of *MsMKK6* and *MsVTI13* (significant SNP located within the gene) as candidate genes for RSA variation. Each set of plots comprises a partial Manhattan plot (top left), the candidate gene structure, an LD heat map (bottom left), and the phenotype values of different haplotypes (right). (E and F) GWAS identification of *MsLBD2* and *MsP4H5* (significant SNP located within the LD block) as candidate genes for RSA variation. Each set of plots comprises a partial Manhattan plot (top left), the distribution of genes within the LD block, an LD heat map (bottom left), and the phenotype values of different haplotypes separated using the top significant SNP (right). In each box, asterisks denote significant differences between haplotype groups (*t*-test, ^*^P < 0.05, ^**^P < 0.01, ^***^P < 0.001, ^****^P < 0.0001). “n” in C–F denotes the number of genotypes in each haplotype group, and each point represents the mean value of six clone plants of each genotype.

By utilizing 1 303 374 high-quality markers (SNPs) with a minor allele frequency ≥ 0.05, and a missing rate < 20.0%, we performed GWAS to explore the genetic basis of root architectural variation using six RSA traits. Sixty significant SNPs (−log_10_(*P*) ≥ 5) were associated with the six root traits, comprising 16, 3, 12, 9, 9, and 11 SNPs for RN, TRD, SRD, RDW, RL, and SRP, respectively (Fig. 3 A, Fig. S3 and Table S4). These markers were distributed on all eight chromosomes (chr), of which chr2, chr3, and chr7 had the least number of markers, with three, three, and two, respectively. Both chr1 and chr4 exhibited the highest number of markers at 11. The remaining chromosomes, chr5, chr6, and chr8, displayed moderately significant SNPs, with eight, seven, and eight SNPs, respectively (Fig. 3A, Fig. S3 and Table S4).

In the regions 50 kb upstream and downstream of the associated SNPs, 308 GWAS candidate genes were identified. All of the identified candidate genes are listed in Table S5. GO enrichment analysis revealed that 128 genes were involved in the biological process pathways (Fig. 3B and Table S6). They were primarily involved in root development biological process pathways such as lateral root morphogenesis (GO:0010102), root morphogenesis (GO:0010015), and root system development (GO:0022622); some biological process pathways related to hormones, such as the gibberellin mediated signaling pathway (GO:0010476), gibberellin biosynthetic process (GO:0009686), hormone biosynthetic process (GO:0042446), hormone-mediated signaling pathway (GO:0009755), and cellular response to hormone stimulus (GO:0032870); and pathways associated with morphogenesis, such as post-embryonic plant organ morphogenesis (GO:0090697), post-embryonic plant morphogenesis (GO:0090698), anatomical structure morphogenesis (GO:0009653), and anatomical structure formation involved in morphogenesis (GO:0048646). The results of the GO enrichment analysis further confirmed the reliability of our GWAS results.

Following the GO enrichment analysis, we functionally annotated genes within the LD blocks of the significant markers. Consequently, we identified 19 high-priority candidate genes whose homologous genes have been confirmed to be involved in regulating the development of the root system in *Arabidopsis* (Table 1). For instance, *MsMKK6* (*Msa.H.0361090*), whose significant SNP (*chr6__123979907*) was located within the gene, was found to be significantly associated with RN (*P* = 8.0 × 10^−6^) (Fig. 3C). Reports indicate that *AtMKK6* positively regulates lateral root formation [56]. *MsVTI13* (a significant SNP located within the gene) was found to be significantly associated with RL (*P* = 1.22 × 10^−6^) (Fig. 3D), and its homologous gene is required for root hair growth in *Arabidopsis* [57]. *Msa.H.0222400* (*MsLBD2*), which is located within a 146.44 kb LD block of significant SNPs on chr4, encodes an LBD protein [16] (Fig. 3E). Additionally, *MsP4H5*, which was associated with SRP (*P* = 4.25 × 10^−6^), was essential for root hair elongation in *Arabidopsis thaliana* [58] (Fig. 3F). The 19 high-priority candidate genes identified in our study, whose homologs of which in *Arabidopsis* have been previously reported to be involved in root development. These results collectively pave the way for more efficient and targeted breeding strategies in alfalfa, potentially leading to varieties that are better suited to varying environmental conditions and agricultural demands.

**Table 1:** List of high-priority candidate genes across the six root traits

**Trait**	**SNP**	**Candidate genes**	**Gene position**	**Arabidopsis best hit**	**Root phenotype**	**Reference**
RN	chr1__64775910	*Msa.H.0041460*	chr1: 64816795–64 822 637	*MAP65* (*PLE*)	Affects root morphology	Muller *et al*. [59]
RN	chr3__95920400	*Msa.H.0171720*	chr3: 95951287–95 966 068	*KIN4A* (*FRA1*)	Root elongation	Zhong *et al.* [60]
RN	chr5__19182230	*Msa.H.0263100*	chr5: 19192567–19 202 527	*KIN14E*	Regulates root growth	Humphrey *et al*. [61]
RN	chr6__123979907	*Msa.H.0361090*	chr6: 123973117–123 979 982	*MKK6*	Lateral root formation	Zeng *et al*. [56]
TRD	chr2__37219057	*Msa.H.0090190*	chr2: 37193037–37 219 680	*AIR9*	Auxin-induced lateral root formation	Neuteboom *et al.* [62]
TRD	chr4__10877367	*Msa.H.0191540*	chr4: 10873000–10 873 592	*AGP30*	Root regeneration	Hengel, Keith Roberts [63]
SRD	chr1__59824300	*Msa.H.0038230*	chr1: 59799246–59 800 171	*LBD29*	Regulate lateral root formation	Okushima *et al*. [16]
SRD	chr1__59824300	*Msa.H.0038220*	chr1: 59792620–59 794 744	*LBD16*	Regulate lateral root formation	Okushima *et al*. [16]
SRD	chr2__77560366	*Msa.H.0112830*	chr2: 77727500–77 730 268	*ACR4*	Restricts formative cell divisions in root	Smet *et al*. [64]
SRD	chr4__67333877	*Msa.H.0222400*	chr4: 67388779–67 391 721	*LBD2*	N/A	N/A
SRD	chr5__34540385	*Msa.H.0272940*	chr5: 34585928–34 588 028	*ARF17*	Major regulator of adventitious rooting	Sorin *et al*. [65]
RDW	chr2__5607797	*Msa.H.0069290*	chr2: 5560083–5 563 388	*WOX9*	Primary root growth	Zhang *et al*. [15]
RDW	chr4__54647717	*Msa.H.0213790*	chr4: 54610408–54 619 353	*TPST*	Regulating post-embryonic maintenance of the root stem cell niche	Zhou *et al*. [66]
RDW	chr8__53077331	*Msa.H.0464970*	chr8: 53120460–53 124 881	*BHLH49*	Regulated by auxin and involved in cell elongation	Radoeva *et al*. [67]
RL	chr4__26327061	*Msa.H.0200690*	chr4: 26346742–26 349 873	*BRN2*	Regulated root cap cellular maturation	Bennett *et al*. [68]
RL	chr4__57176292	*Msa.H.0215210*	chr4: 57134722–57 135 424	*EXPA10*	Cell elongation	Samalova *et al*. [69]
RL	chr7__16119248	*Msa.H.0379970*	chr7: 16116968–16 124 075	*VTI13*	Root hair growth	Larson *et al*. [57]
SRP	chr1__33054664	*Msa.H.0023570*	chr1: 33014781–33 018 841	*XLG1*	Regulate root morphogenesis	Ding *et al.* [70]
SRP	chr5__17133632	*Msa.H.0261570*	chr5: 17097186–17 102 073	*P4H5*	Root hair growth	Velasquez *et al*. [58]

RN, root number; TRD, taproot diameter; SRD, secondary root diameter; RDW, root dry weight; RL, root length; SRP, secondary root position.

### The role of favorable haplotypes in alfalfa breeding

**Figure 4:**
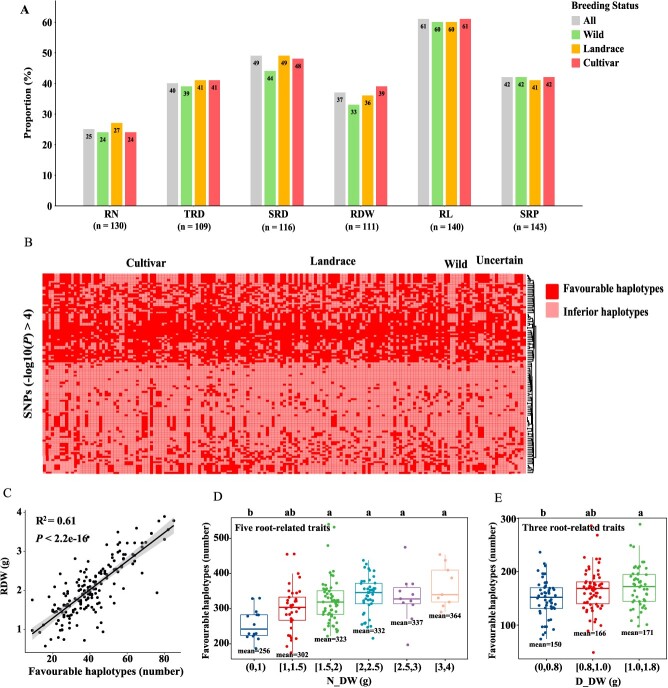
Role of favorable haplotypes in alfalfa breeding progress. (A) The proportion of favorable haplotypes in different improved status materials across the six root-related traits. The “n” below each trait indicates the number of associated SNPs (−log_10_(*P*) ≥ 4), whose phenotype has significant differences (pairwise.t.test, *P* < 0.05) between the haplotype group. (B) Distribution of favorable haplotypes related to RDW in alfalfa materials from different improved statuses: cultivar (*n* = 70), landrace (*n* = 70), wild (*n* = 13), and uncertain (*n* = 18). Further details of the distribution of favorable haplotypes for the six RSA traits are included in Figs S4 and S5 and Table S7. (C) The number of favorable haplotypes corresponding to root dry weight (RDW) in our association panel. Linear regression was used for the analysis, and the coefficient of determination (*R*^2^) and *P*-value of the resulting equation were provided. The *x*-axis represents the number of favorable haplotypes identified by GWAS. The *y*-axis represents the weight of the dry root. Additional results for the remaining five RSA traits are shown in Fig. S6. (D, E) Number of favorable haplotypes in alfalfa materials with different forage dry weights (N_DW and D_DW). The x-axis shows the group divided according to the forage dry weight. The y-axis is the total number of favorable haplotypes related to the appropriate root system architecture identified by the GWAS. Five RSA traits (RN, TRD, SRD, RDW, and RL) related to N_DW, and Three RSA traits (RN, TRD, and RL) related to D_DW were selected for this analysis. The lower and upper boundaries represent the 25th and 75th percentiles, respectively. The middle horizontal line represents the median value. Different letters indicate significant differences at *P* < 0.05 (Tukey's all-pair comparisons). RN, root number; TRD, taproot diameter; SRD, secondary root diameter; RDW, root dry weight; RL, root length; SRP, secondary root position; DW, dry weight. “N_” and “D_” symbolize normal and drought conditions, respectively. N_DW and D_DW represent forage dry weight under normal and drought conditions, respectively.

SNPs associated with root phenotypes in GWAS were used to study changes in favorable haplotype frequencies over the course of modern alfalfa breeding. Among the six root-related phenotypes, 130 (RN), 109 (TRD), 116 (SRD), 111 (RDW), 140 (RL), and 143 (SRP) SNPs exceeded the threshold line (−log_10_(*P*) ≥ 4) and showed significant phenotypic differences between haplotypes (Table S7). These markers were used for subsequent analyses. In the association panel, the proportions of these favorable haplotypes in the six traits were 0.25, 0.40, 0.49, 0.37, 0.61, and 0.42 for RN, TRD, SRD, RDW, RL, and SRP, respectively (Figs S4 and S5 and Table S7). Except for RDW, the proportion of favorable haplotypes in the different breeding status materials remained largely unchanged (Fig. 4A). For RDW, the proportion of favorable haplotypes in the cultivars was marginally higher than in wild materials, at 0.39 compared to 0.33 (Fig. 4A and B). Favorable haplotypes have not been fully utilized in alfalfa breeding for all six phenotypes.

The correlation between each root-related trait and the number of favorable haplotypes in the association panel was also analyzed. The results showed significant correlations between six RSA traits and the number of favorable haplotypes, with the coefficient of determination (*R*^2^) ranging from 0.61 to 0.75 (Fig. 4C and Fig. S6). For instance, the number of favorable haplotypes and RDW had the lowest R^2^ of 0.61, implying that as the number of favorable haplotypes increased, the dry weight of roots tended to increase (Fig. 4 C). The remaining five traits had an R^2^ of 0.68 (*P* < 2.2e^−16^, RN), 0.68 (*P* < 2.2e^−16^, TRD), 0.61 (*P* < 2.2e^−16^, SRD), 0.75 (*P* < 2.2e^−16^, RL), and 0.65 (*P* < 2.2e^−16^, SRP) (Fig. S6). Overall, alfalfa plants with more favorable haplotypes had more optimal root phenotypes, indicating that the SNPs identified in our study can be used for genetic improvement in alfalfa root breeding programs.

To further verify the application potential of these SNPs in alfalfa breeding, we calculated the relationship between the number of favorable haplotypes and forage yield. The correlation between the number of favorable haplotypes of five traits (RN, TRD, SRD, RDW, and RL) and forage yield was calculated because they had significant correlation coefficients with N_DW (Fig. S7). We divided the associated panel into six groups based on dry weight (N_DW). As the dry weight increased, the number of favorable haplotypes increased significantly, with 256, 302, 323, 332, 337, and 364, respectively (Fig. 4 D). More favorable alleles accumulated in higher-yield materials. Similar results were observed under drought conditions (Figs 4E and S8). Combined with the above results, the RSA trait-related markers identified in this study could provide valuable genetic resources for the genetic improvement of alfalfa, with great potential for application in alfalfa breeding projects.

### GP of six RSA traits using GWAS-derived SNP markers

**Figure 5:**
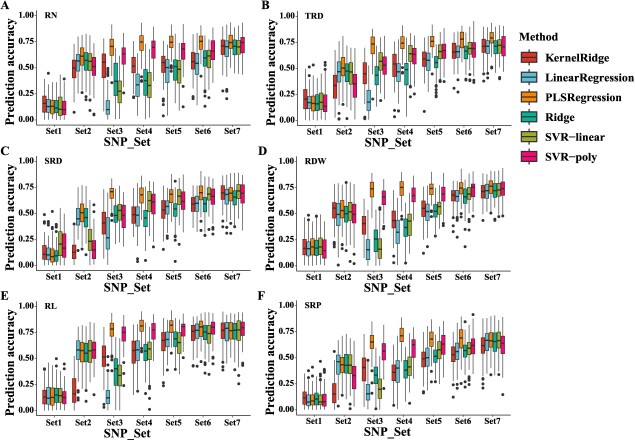
Genomic prediction accuracy of six machine learning models among seven SNP sets (Set1–7) for each root-related trait. (A–F) Genomic prediction accuracy for RN, TRD, SRD, RDW, RL, and SRP, respectively. Set1 contained 306 411 SNPs filtered by LD. Set2–Set7 were obtained using GWAS. SNPs with -log_10_(*P*) ≥ 5 (Set2), SNPs with −log_10_(*P*) ≥ 4 (Set3), top 300 association markers (Set4), top 500 (Set5), top 1000 (Set6), and top 5000 (Set7). RN, root number; TRD, taproot diameter; SRD, secondary root diameter; RDW, root dry weight; RL, root length; SRP, secondary root position.

This study utilized nine SNP sets and six GP models, yielding 54 combinations for each RSA trait. The average GP accuracies (*r*) from the 100 runs for each combination were listed in Table S8. When employing all LD_filtered markers for GP, all models exhibited extremely low accuracy across the six RSA traits, with PA mean values ranging from 0.11 (SRP) to 0.18 (TRD and RDW) (Fig. 5 and Table S8). When employing GWAS-derived SNP sets, the accuracy of each method improved significantly for the six RSA traits. In general, the accuracy of each model increased as the number of GWAS-derived markers increased, and when the number of associated markers reached 5000 (set7), the accuracy of each model stabilized (Fig. 5, Table S8). Some methods experienced reduced prediction accuracy due to using of SNP set8 and set9 (Table S8). Across all six traits, it is noteworthy that the PLSRegression method demonstrated higher accuracy with fewer GWAS-derived markers. When employing SNP set3 (−log_10_(*P*) ≥ 4), the PA of the PLSRegression method amounted to 0.69, 0.72, 0.69, 0.73, 0.76, and 0.64 for RN, TRD, SRD, RDW, RL, and SRP, respectively (Fig. 5 and Table S8). Concurrently, the PLSRegression method achieved the highest prediction accuracy in RN, TRD, RDW, RL, and SRP, amounting to 0.74 (set6), 0.79 (set7), 0.75 (set7), 0.80 (set4), and 0.71 (set4), respectively. For SRP, the PLSRegression method attained the second highest accuracy (0.69), behind only the SVR-linear method (0.70) (Fig. 5 and Table S8). Compared to the very low average GP in SNP set1, model accuracy improved significantly using associated SNP markers, improving accuracy to between 0.70 (SPR) and 0.80 (RL) across the six root traits. All the aforementioned results indicate that all SNPs filtered by LD cannot be directly used for six RSA traits GP of the six RSA traits, but utilizing associated SNP markers will increase selection efficiency.

## Discussion

### The feasibility of improving alfalfa using markers associated with RSA traits

Alfalfa is an important perennial forage legume. However, there has been slow progress in terms of yield improvements. The yield benefit derived from the alfalfa breeding program amounted to approximately 0.25% per year [71]. Many studies have highlighted the potential benefits of improving root system-related traits and have reported that a more developed RSA enhances crop yield and stress resistance [3, 17, 72–74], thus contributing to higher economic returns. The RSA has evolved to adapt to planting needs during the alfalfa breeding process. These changes in root structure are indirectly influenced by human selection of above-ground phenotypes [27, 28]. The correlation between root traits and above-ground phenotypes is important for the indirect selection of root traits in alfalfa breeding. Here, we provided evidence that RSA traits significantly correlated with yield-related phenotypes (Fig. 2), a finding consistent with previous studies [19, 23]. Surprisingly, RL showed a negative correlation with D_DW, with a correlation coefficient of −0.21 (*P* < 0.01). This appears to be constrained by the potting environment, where root structures with a greater spread exhibit a larger surface area for water absorption than longer roots. Quantitative trait loci mapping and GWAS are essential tools in molecular breeding programs, playing an irreplaceable role in identifying reliable and stable loci and functional genes controlling quantitative traits [37, 38, 75, 76]. Using GWAS, we identified numerous SNPs linked to RSA traits, with the count of favorable haplotypes correlating with alfalfa yield (N_DW and D_DW). High-yield materials harbored more favorable haplotypes (Fig. 4). Additionally, our analysis of haplotype distribution revealed that these favorable haplotypes were not strongly selected in modern alfalfa breeding programs. (Fig. 4). This could be related to the lower yield benefits observed in modern alfalfa cultivars [71]. Although these favorable haplotypes for RSA traits have not experienced strong selection, we observed an increase in the proportion of favorable haplotypes associated with RDW from 0.33 in wild materials to 0.39 in cultivated materials. The slow increase of favorable haplotypes might be due to their higher correlation with yield-related traits. The favorable haplotypes identified in our research have significant potential applications, making them worthwhile targets for future alfalfa root genetic improvement, and can be leveraged in molecular breeding to improve the alfalfa root system and yield.

### Candidate genes for RSA traits

Although numerous genetic studies have been conducted on the agronomic traits of alfalfa, few genes controlling alfalfa RSA have been identified. Here, we analyzed the root systems of adult alfalfa plants and identified 60 SNPs and 308 candidate genes. Additionally, we combined the GWAS results with GO enrichment analysis, annotating 19 high-priority candidate genes (Table 1), whose homologous genes have been reported to be related to root development in *Arabidopsis*. These genes gave confidence to our GWAS results and represented high-quality candidate genes for alfalfa RSA traits. Several genes regulate root development through the auxin signaling pathway, which plays an important regulatory role in plant root development [5, 14, 77]. For example, we identified three *lateral organ boundary domain* genes (*LBD2*, *LBD16*, and *LBD29*) involved in the root growth process of the auxin signaling pathway [16, 78, 79]. *LBD16* and *LBD29* are specifically activated by two auxin response factors (*ARF7* and *ARF19*), which regulate lateral root formation [16]. Interestingly, we discovered an auxin response factor gene, *ARF17*, in this study. The identified genes can be leveraged for future root genetic improvements and have the potential to limit yield losses caused by environmental extremes, such as drought. Despite the success of GWAS in identifying genes associated with RSA traits, characterizing the functions of these genes in regulating alfalfa root development poses a significant challenge. Detailed characterization of these genes, although fascinating, is beyond the scope of this study. However, in subsequent studies, we will continue to explore the relationship between these genes and root development in alfalfa.

### The improved efficiency of GP using GWAS-derived markers combined with machine learning algorithms

Traditional alfalfa breeding methods are primarily based on recurrent phenotype selection [80], which is constrained by the accuracy of phenotype statistics. However, gathering statistics on alfalfa root phenotypes under field conditions is challenging, resulting in low and unpredictable selection efficiency for alfalfa root improvement based on phenotypic selection. GP is an efficient method for predicting crop trait performance utilizing genomic markers [36]. Making predictions based on genotypic information is a more cost-effective option for phenotypes that are difficult to collect, such as RSA traits. To date, GP for root traits has been reported in several crops [81–83]. The use of GWAS-derived SNP markers in GP has been proven advantageous [37, 38]. Shi et al. (2022) reported that significant GWAS-derived SNP markers could increase PA with a high *r* value up to 0.84, much higher than PA using randomly selected SNP sets [38]. Our previous study demonstrated that combining GWAS-related markers with machine learning can improve fall dormancy prediction accuracy in alfalfa [37]. This study employed nine SNP sets and six machine learning methods to perform the GP of six root traits. The average PA values in SNP Set1, which was filtered by LD, ranged from 0.11 to 0.18. However, the highest average PA values for each trait ranged from 0.70 to 0.80 when GWAS-derived markers were employed (Fig. 5). Using a similar number (3000 SNPs) randomly chosen from SNP Set1 did not enhance the accuracy (results not shown). This suggested that a random set of SNPs with no association with the RSA traits was ineffective for enhancing PA for the RSA trait, whereas using associated SNP markers can improve selection efficiency. Although performing GP in the same association panel utilizing GWAS-related SNP markers may show inflated prediction accuracy, the feasibility of this method has been validated for various traits in several crops [38, 84, 85]. Consequently, leveraging GWAS-derived markers and machine learning for GP would be beneficial for the molecular breeding of root-related traits in alfalfa and other quantitative traits in different crops.

Although our study opens new doors for alfalfa breeding, further research is needed to apply these findings to real-world agriculture. We should recognize substantial differences between the root system phenotypes of alfalfa grown in a greenhouse and alfalfa cultivated in the field [11]. Furthermore, we did not measure the fibrous root phenotype, which is important for alfalfa herbage yield [23, 86] because of the challenges associated with its separation from the soil. As a perennial crop, the root system of alfalfa undergoes dynamic changes over different years, depending on environmental and management factors. Therefore, despite the valuable insights that our research offers for the genetic improvement of alfalfa root traits, exploration of the developmental process of the alfalfa root system and identification and characterization of its related genes will require considerable effort. Future research should focus on the functional validation of these genes and explore gene–environment interactions to understand better how these genetic factors perform in diverse agricultural settings.

In summary, we phenotyped alfalfa root structures in a greenhouse, revealing a significant correlation between the root architecture and yield traits. A GWAS identified 60 SNPs (−log10(P) ≥ 5) and 19 candidate genes related to RSA. Haplotype analysis suggested that alfalfa yield can be improved by aggregating favorable haplotypes. Moreover, combining GP with machine learning and GWAS-derived markers achieved a prediction accuracy of 0.70 to 0.80 for root traits. This study demonstrated the potential of harnessing a combination of GWAS and GP for the genetic improvement of RSA traits in alfalfa, which could pave the way for mitigating the trade-off between alfalfa yield and stress resistance.

## Methods

### Population materials

An alfalfa association panel of 171 genetically diverse materials was prepared, including 13 wild, 70 landrace, 70 cultivar, and 18 uncertain materials collected from six continents, representing the global genetic diversity of alfalfa (Table S1). The association panel included 24 accessions from the China National Grass Seed Resource Interim Bank, and 144 accessions from the online database of the U.S. National Plant Germplasm System (https://npgsweb.ars-grin.gov/gringlobal/search), and three commercial alfalfa varieties (Table S1). The 171 materials were grown under field conditions at the research station of the Ningxia Academy of Agricultural and Forestry Sciences in Yinchuan, Ningxia Province (38.21°N, 106.22°E).

### Growth conditions and phenotyping

These materials were cut and propagated in a greenhouse in May 2022. Uniform clonal plants were selected and transferred to pots between August 12 and 14. The dry weight of the commercial potting soil (Hengxian substrate) in each pot was approximately 300 g, a nutrient-rich peat potting mix. Each genotype was transplanted into nine pots, with two clonal plants per pot, for a total of 18 plants. After two cuttings, we selected six pots of plants with consistent growth for further experiments and phenotypic collection to ensure the accuracy of the phenotypic data. We divided these six pots into two groups, one for the control treatment and one for the drought treatment, with three replicates per group. Each pot was replicated once. The control group was watered to ensure the soil moisture content reached 75 ± 5%, whereas the treatment group reached 33 ± 2%. The remaining three pots were used for drought pretreatment to select marker materials. In the pretreatment process, two materials (PI 435232 and PI 435231) had the fastest regeneration rates, consumed the most water, and experienced the most rapid decline in soil moisture content. These two materials were used as markers. Watering was started when the soil moisture content of the two materials in the control group dropped to approximately 35% during the experimental process. Watering was initiated when the leaves of the two plants in the drought treatment group started to wilt. The moisture control process was undertaken by weighing the pots to estimate the required water amount. Next, we used a Delta T ML3 soil moisture analyzer to measure and calibrate the soil's water content, ensuring consistent moisture levels with each application.

Root phenotypes were measured using materials from the control group. The roots were gently shaken to remove most of the attached soil. The roots were then soaked in water, and the remaining soil and fibrous roots were removed. We measured six alfalfa RSA-related phenotypes, including root number (RN), taproot diameter (TRD), secondary root diameter (SRD), root dry weight (RDW), root length (RL), and secondary root position (SRP), using ImageJ software to measure TRD and SRD from the JPEG images (Table S2). Five yield-related traits were also collected to investigate the relationship between alfalfa RSA traits and yield. Under normal (N) conditions, these traits included regeneration plant height (N_RPH), branch number (N_BN), plant height (N_PH), leaf area (N_LA), and dry weight (N_DW). Relevant phenotypes under drought conditions were also collected, where D_DW represents the dry weight under drought (D) conditions. After 21 days from cutting, the height of regenerated plants was measured, whereas other phenotypic traits were evaluated during the budding stage, precisely when visible flower buds emerged.

### Phenotype analysis

The mean value of each genotype was calculated for the six clonal plants and used for basic statistical analysis of the phenotypes, correlation analysis, cluster analysis, and principal component analysis (PCA). The correlation between root and yield under both normal and drought conditions was estimated using Pearson’s correlations between pairs of traits with the “cor” function in R. A correlation heatmap was created using the R package corrplot [44]. The cluster analysis of six root traits was performed using ChiPlot (https://www.chiplot.online/) with the “complete” method. PCA was performed using the R function of “prcomp” and the results were displayed using the R package factoextra [45].

### Genotyping and SNP calling

Total DNA was extracted using the CWBIO Plant Genomic DNA Kit (CoWin Biosciences, Beijing, China) according to the manufacturer's protocol and sequenced on the BGI-Shenzhen DNBSE platform (BGI, Shenzhen, China). Approximately 36 Gb of raw sequencing data was obtained for each genotype. The adapters from the raw data were subsequently removed and low-quality sequences were filtered using Trimmomatic (v.0.39) [46]. BWA-MEM [47] was used to align clean sequencing data with the reference genome of the zhongmu-4 haplotype [43], which represents a merged genome (0.79 Gb) of four homologous chromosomes. SAMtools (v1.13) was used to filter multiple alignments and low-quality sequences, and the filtered bam files were obtained and sorted [48]. PCR duplicates were marked using the MarkDuplicates function of the Picard package (v2.23.0). The resulting bam files were labeled for variant calling with GATK HaplotypeCaller (v4.2.3.062). We applied the following parameters to filter the SNPs: QD < 2.0 || FS >60.0 || MQRankSum < − 12.5 || ReadPosRankSum < − 8.0 || SOR >3.0 || MQ < 40.0. Two subsets of the alfalfa SNPs were defined as follows: (i) a base SNP set of bi-allelic SNPs was created using VCFtools (v0.1.16) [49] by removing SNPs with >20% missing calls and minor allele frequency (MAF) < 0.05%, and (ii) a linkage disequilibrium (LD) filtered SNP set was derived from the base SNP set using PLINK (v1.90b6.21) [50], in which SNPs were removed by LD pruning with a window size of 100 SNPs, window step of 50 SNPs, and an *r*^2^ threshold of 0.2. The missing genotypes of the LD_filtered SNP set were imputed using Beagle software with default parameters [51]. For GWAS, the basic SNP set was used, whereas for GP, the imputed LD_filtered SNP set was employed.

### GWAS of root traits and candidate gene annotations

We used the R package GAPIT [52] with the Bayesian-information and linkage-disequilibrium iteratively nested keyway (BLINK) model [53] to conduct GWAS for six RSA traits via 1 303 374 high-quality SNP markers. For the association analysis in GAPIT, the first five PCAs were used as covariates with the following parameters: PCA.total = 5. The Genetic Type I error calculator (GEC, http://statgenpro.psychiatry.hku.hk/gec/) was used to evaluate the significance level of SNP *P*.values in GWAS. The suggestive threshold was 5.89. Given the limited number of significant markers at this threshold (only 13 SNPs for six root traits), and to balance false positives and false negatives, we conservatively chose −log_10_(*P*) = 5.0 as a threshold for calling significant associations. The GWAS results were visualized using Manhattan plots generated using the R package CMplot. Candidate genes were obtained by searching for genes within 50 kb upstream and downstream of each significantly associated SNP based on the alfalfa genome sequence. TBtools [54] was used to perform Gene Ontology (GO) enrichment analysis on these candidate genes in order to validate the reliability of the GWAS results and narrow down the candidate gene selection. Genes enriched in root development, hormones, and morphogenesis pathways were further analyzed in EnsemblPlants (https://plants.ensembl.org/index.html) to identify the best *Arabidopsis* match gene annotations. Genes annotated as related to root development were considered high-priority candidates.

### Analysis of favorable haplotypes by GWAS

At a threshold of 5, only a few loci were identified across the six traits, with only three SNPs detected in TRD. In this study, we utilized trait-associated SNPs (with a significance threshold of −log_10_(*P*) ≥ 4) to investigate the frequency distribution of favorable haplotypes within the association panel and their potential application in alfalfa breeding programs. Subsequently, SNPs showing significant phenotypic differences between haplotypes were analyzed using the “pairwise.t.test” function in R. Haplotypes associated with appropriate RSA traits, which were associated with higher yields, were considered favorable. Previous correlation analyses identified genotypes exhibiting greater RNs, larger taproot and lateral root diameters, heavier root dry weights, and shorter root lengths as favorable haplotypes.

### GP with different models

The GP was conducted using six machine learning methods implemented in the Python package sklearn (https://scikitlearn.org/stable/): ordinary least squares linear regression (LinearRegression), a form of regularized linear regression (PLSRegression), ridge regression regularized by L2-norm (Ridge), a method combining ridge regression and classification with the kernel trick (KernelRidge), and two models based on the concept of support vector machines (SVR-linear and SVR-poly). We performed GP for six RSA traits using a 5-fold cross-validation, considering both the number of SNPs and GP models. The association panel was randomly divided into 80% training and 20% testing genotypes to generate 100 samples. Predictive models were constructed using nine SNP sets (Set1–Set9). Set1 comprised 306 411 SNP markers filtered by LD, whereas the other sets were obtained from GWAS. Sets 2–9 were defined based on significance thresholds of -log10(P) ≥ 5, −log10(P) ≥ 4, and the top association markers (300, 500, 1000, 5000, 10 000, and 20 000), respectively. To assess model performance, we calculated Pearson’s correlation coefficient (*r*) between the estimated values of the training genotypes and the observed phenotypes. Finally, a distribution plot of *r* values was generated from the 100 test results using the R package ggplot2 [55].

## Supplementary Material

Web_Material_uhae271

## Data Availability

All RAD raw sequence data were uploaded to the National Genomics Data Center (NGDC, https://bigd.big.ac.cn/) under BioProject PRJCA018485 and NCBI Sequence Read Archive with Bioproject ID: PRJNA995892. The datasets used and analyzed during the current study are available from the corresponding author on reasonable request.
