# Supplementary figures and images for: The whole-genome dissection of root system architecture provides new insights for the genetic improvement of alfalfa (Medicago sativa L.)
Source: Hortic Res. 2024 Nov 4;12(1):uhae271. doi: 10.1093/hr/uhae271 (PMC11725648; doi:10.1093/hr/uhae271)

**A**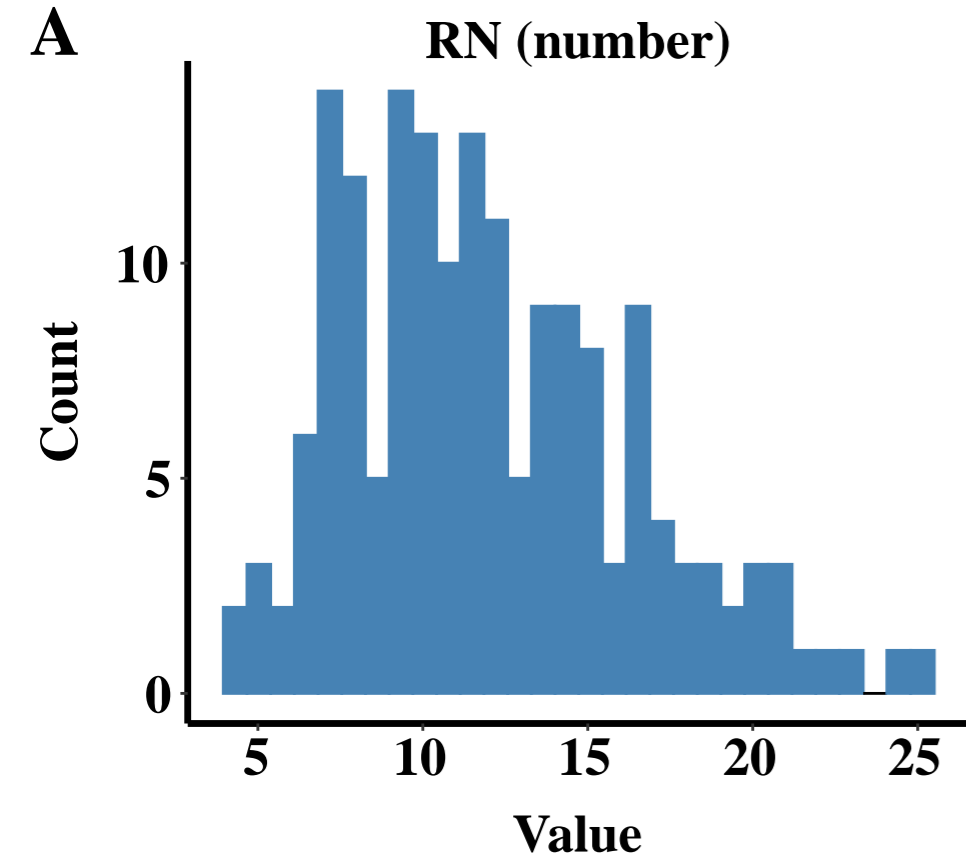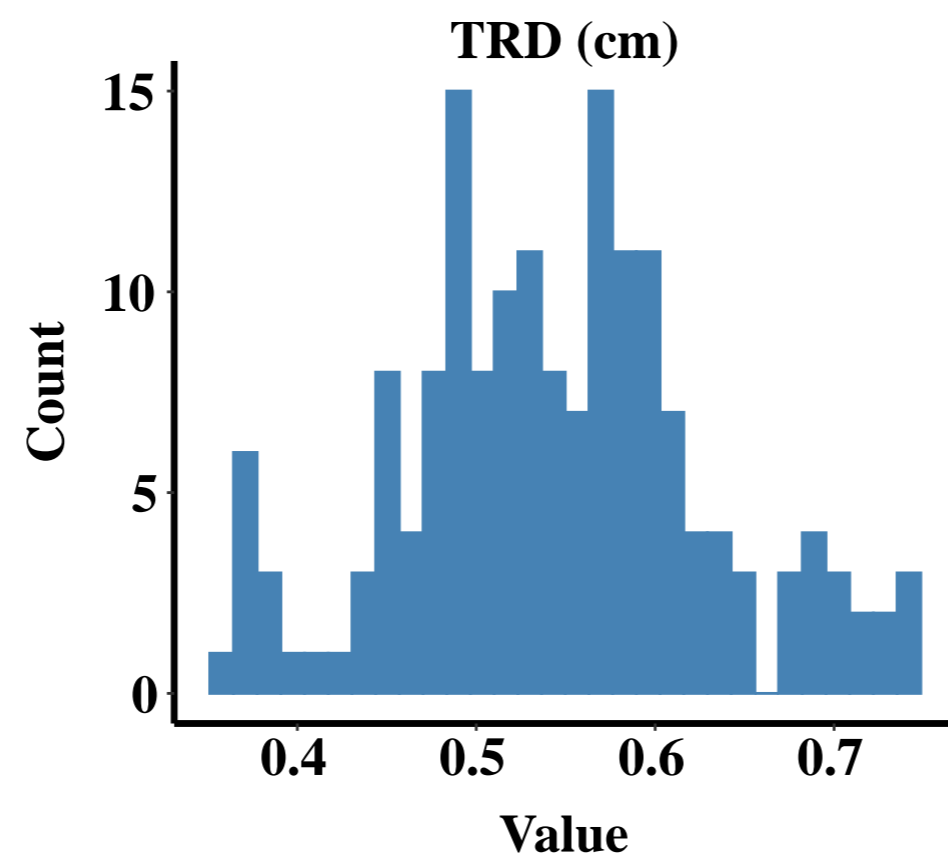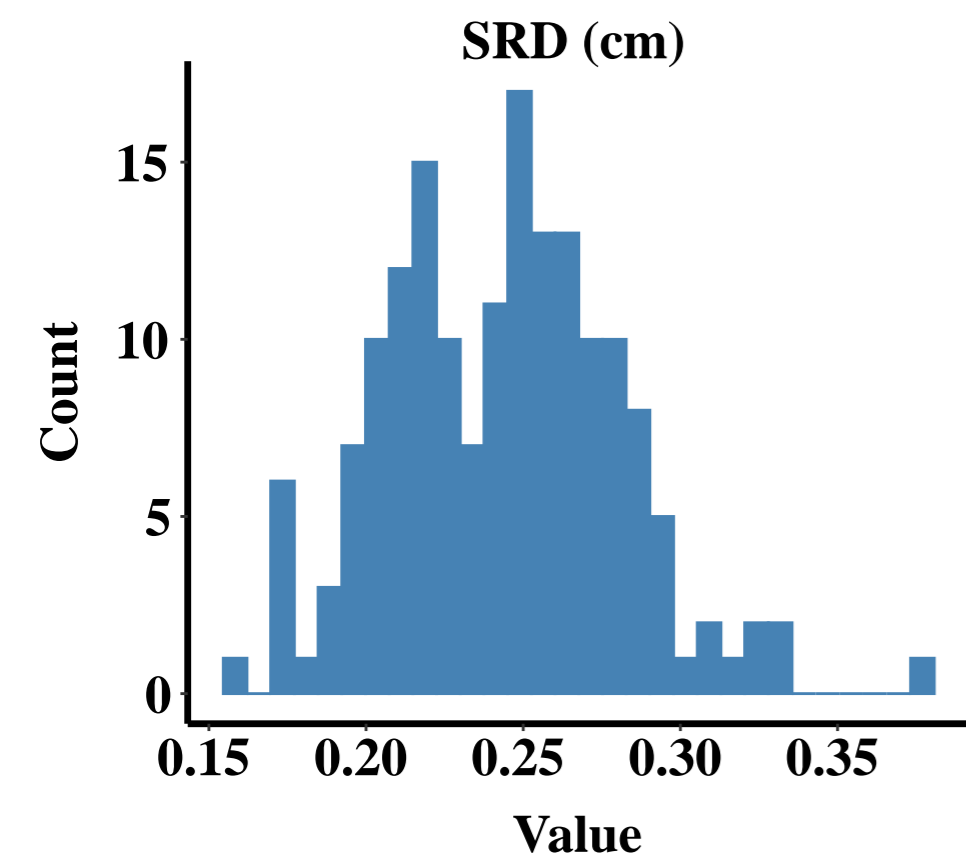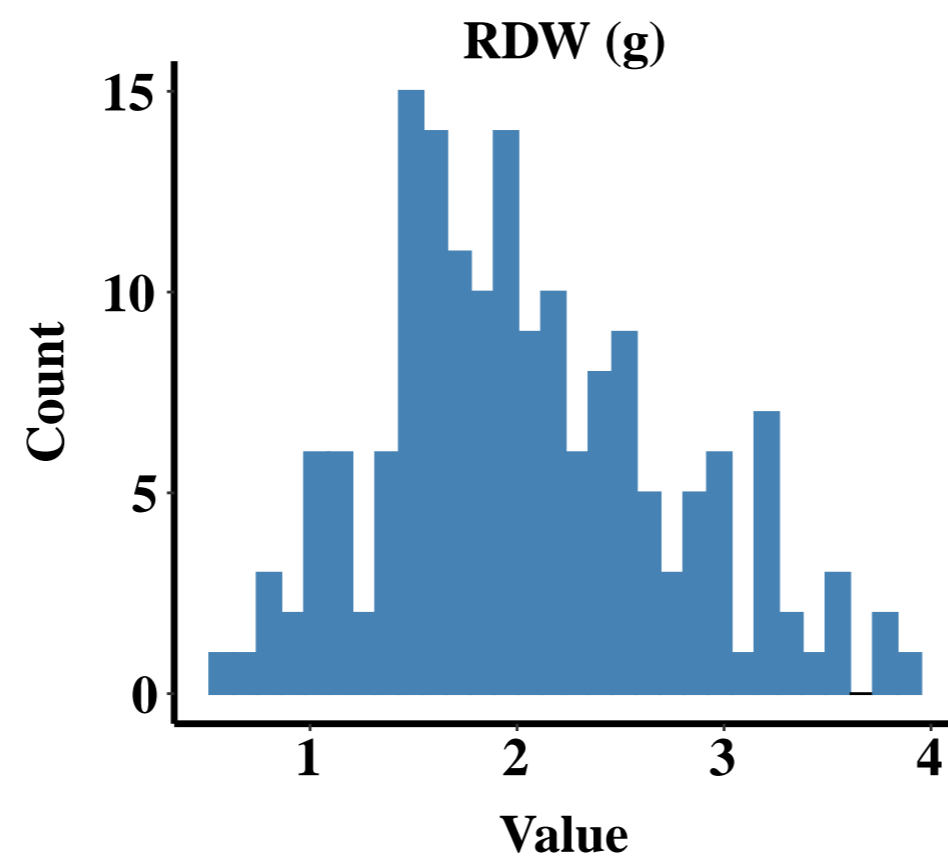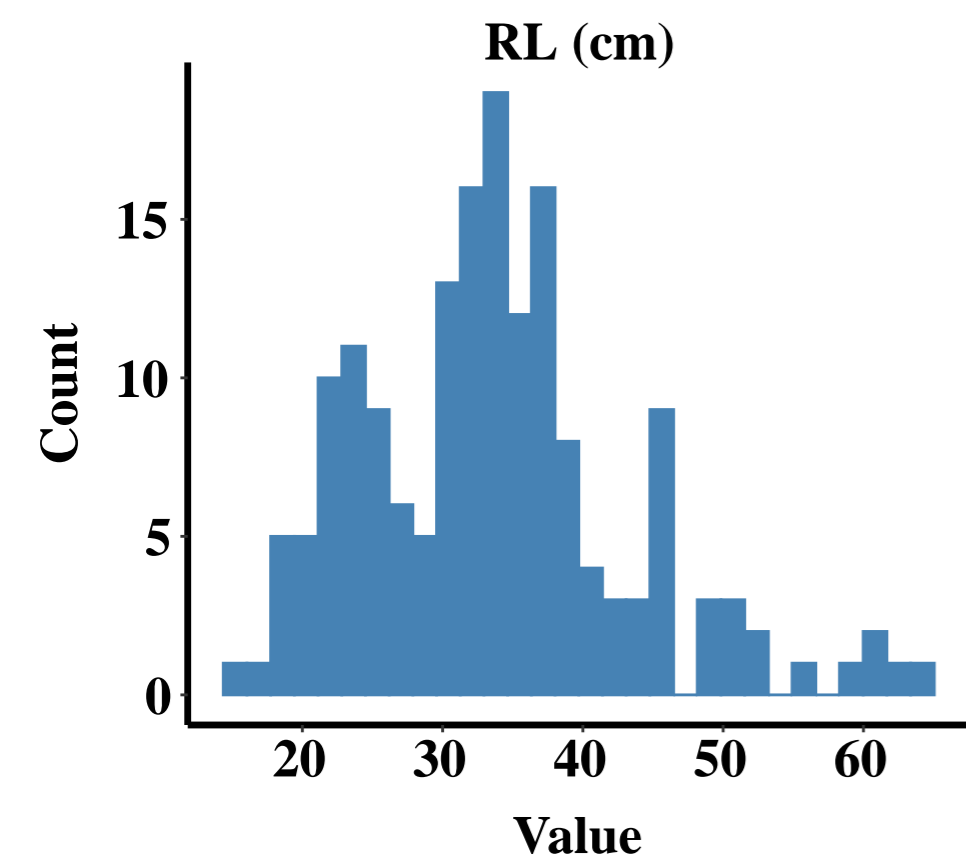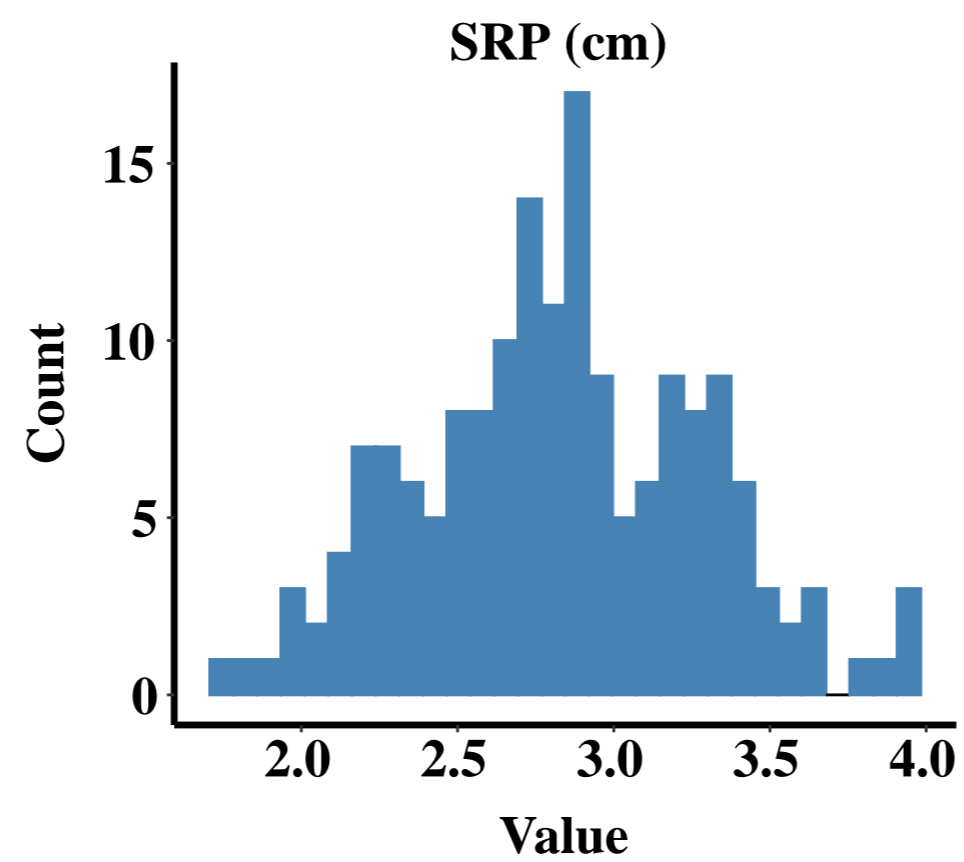**B**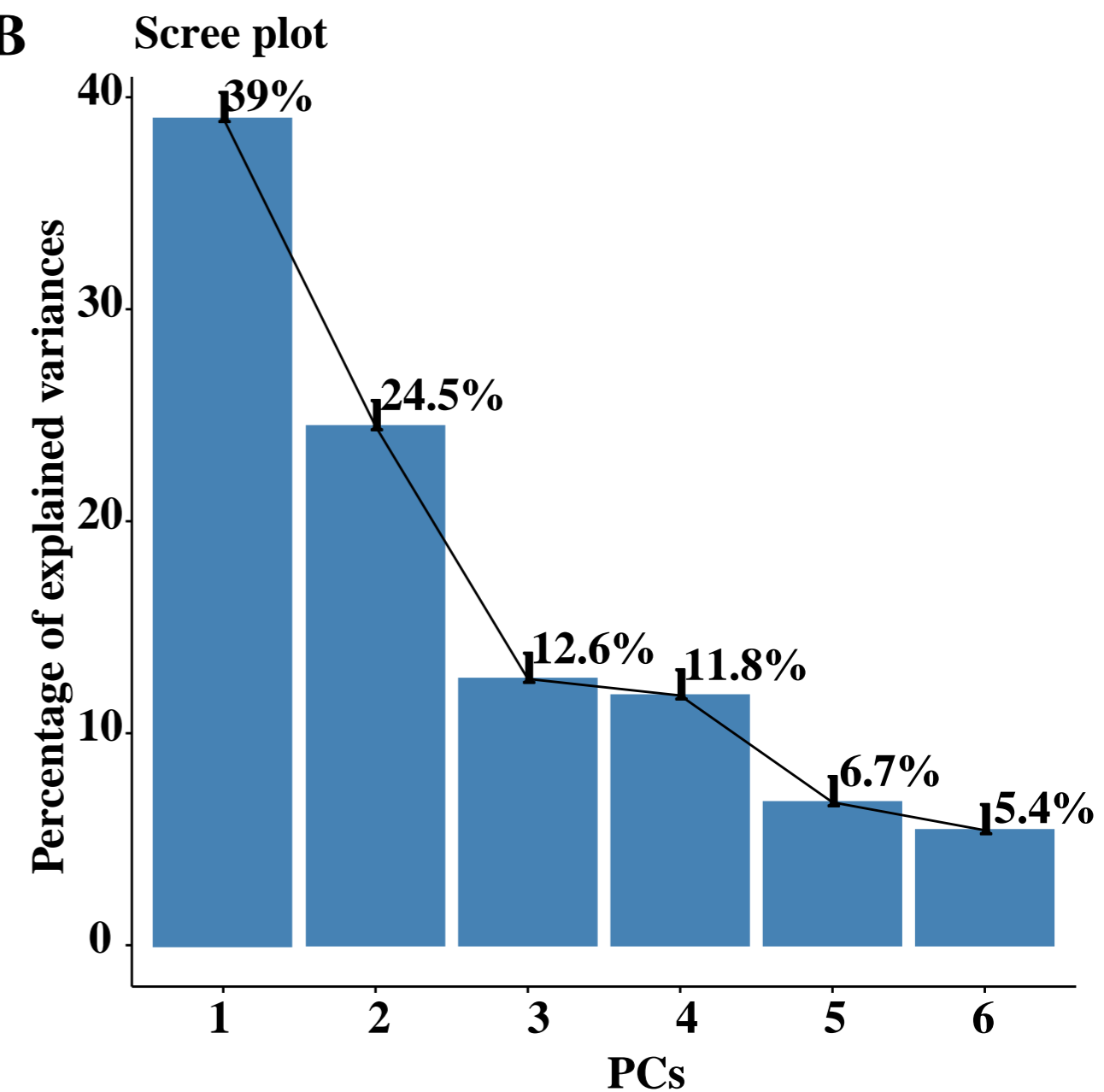**C**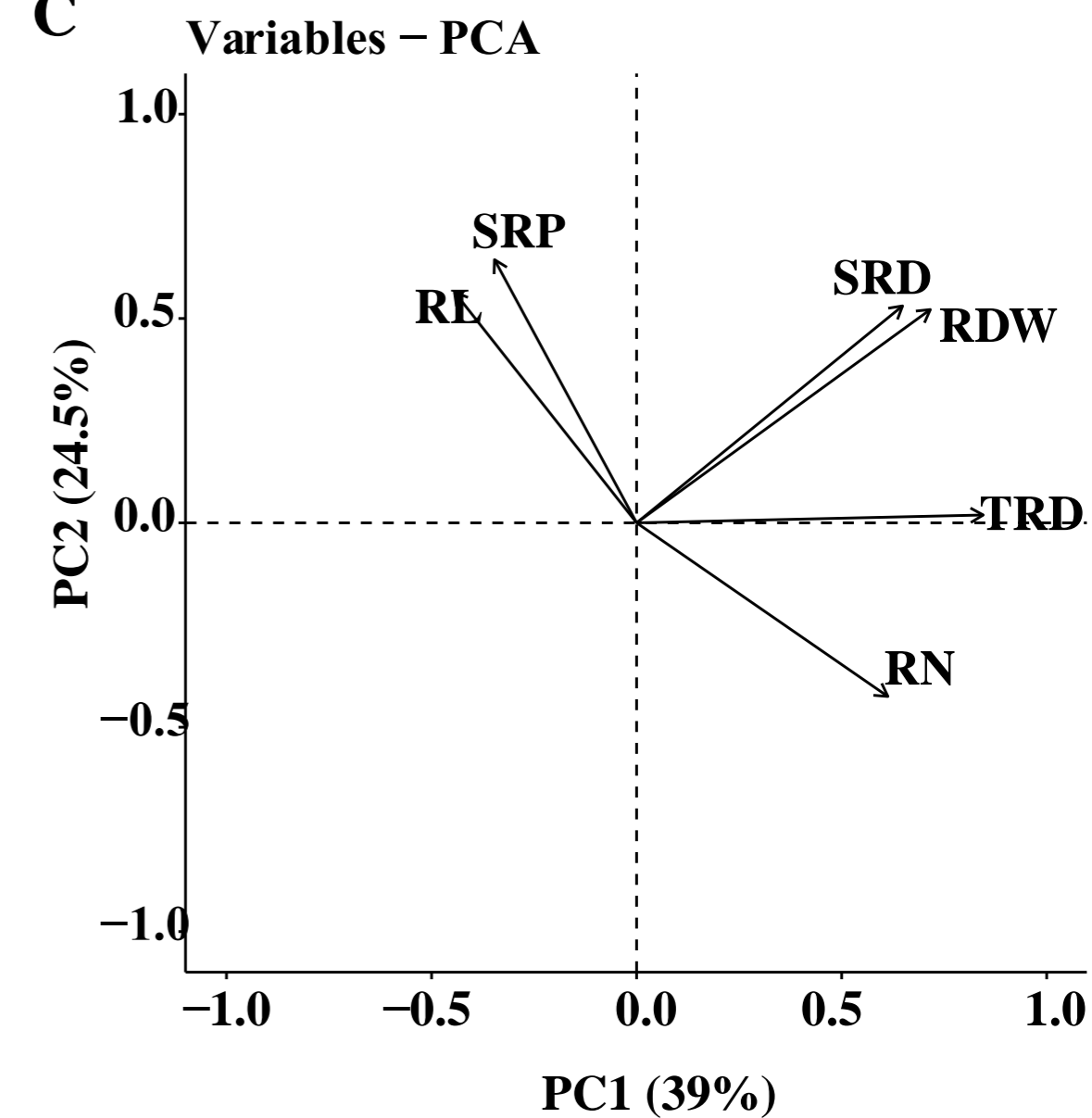

Supplement: Web_Material_uhae271 [file web_material_uhae271.zip › Fig S1.pdf]

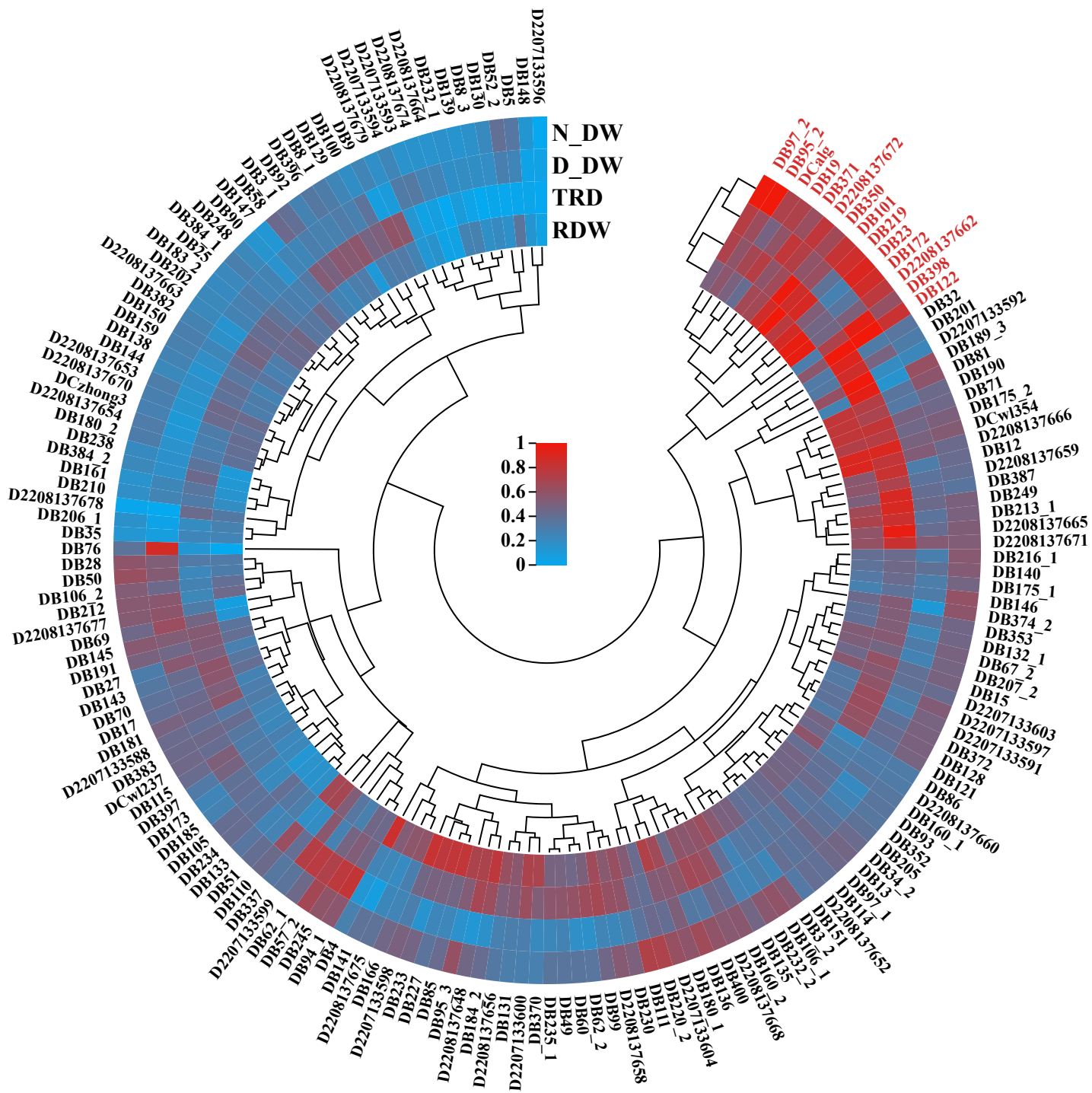

Supplement: Web_Material_uhae271 [file web_material_uhae271.zip › Fig S2.pdf]

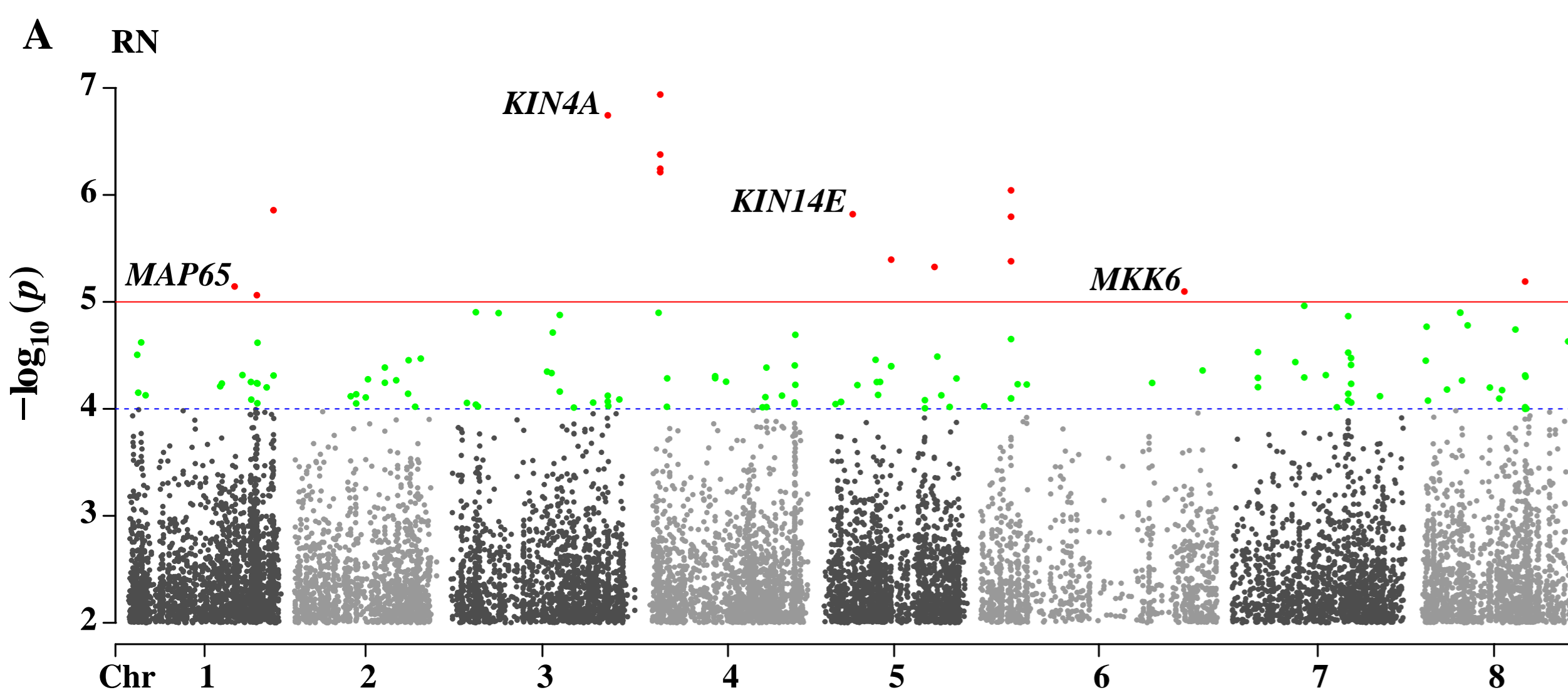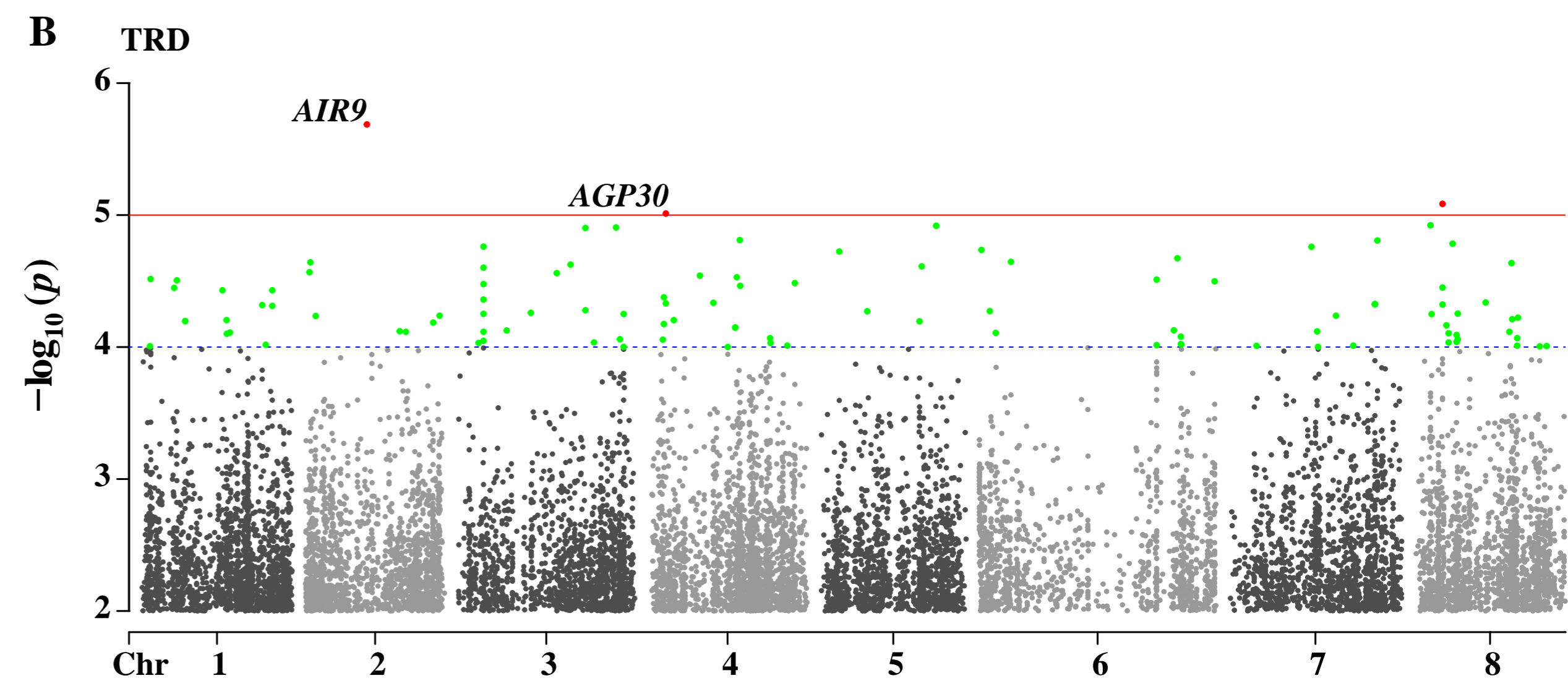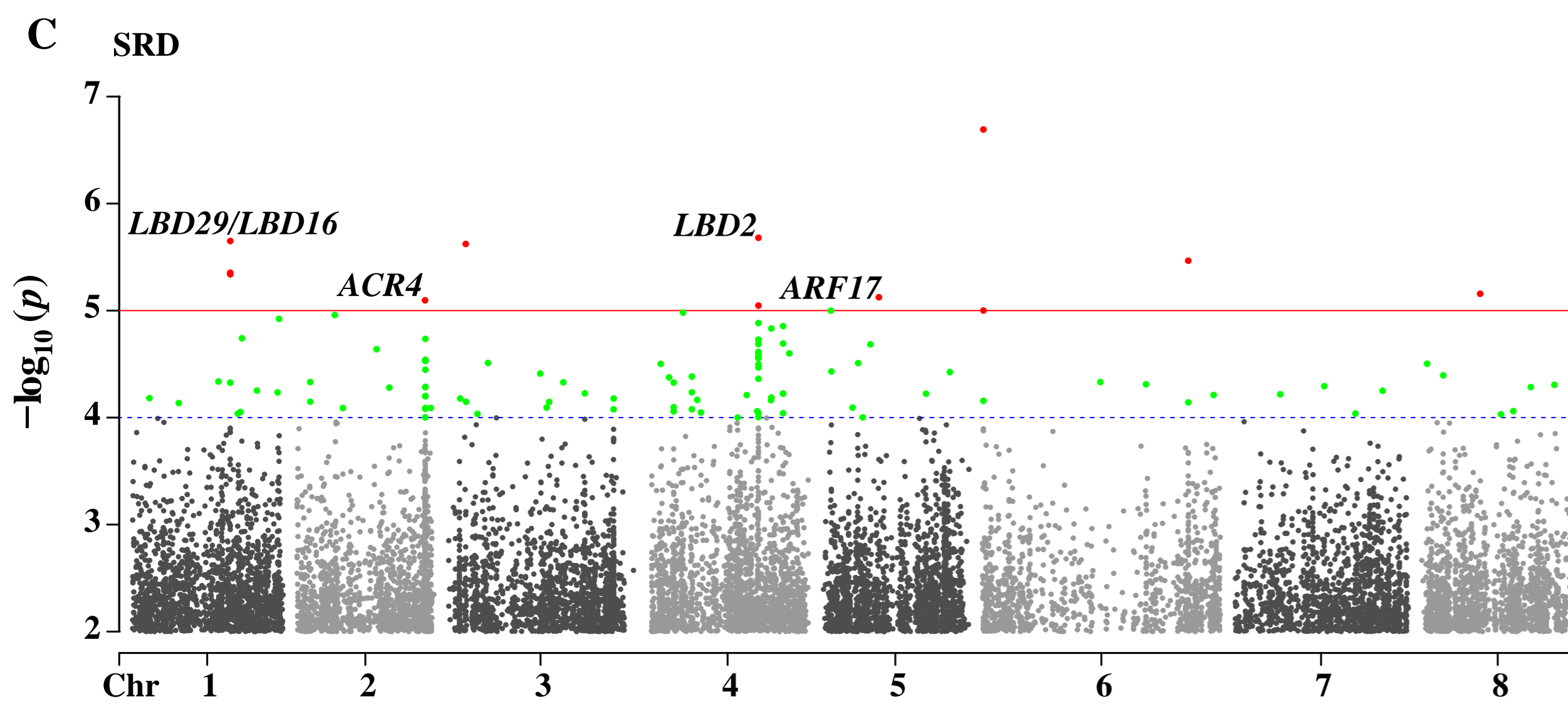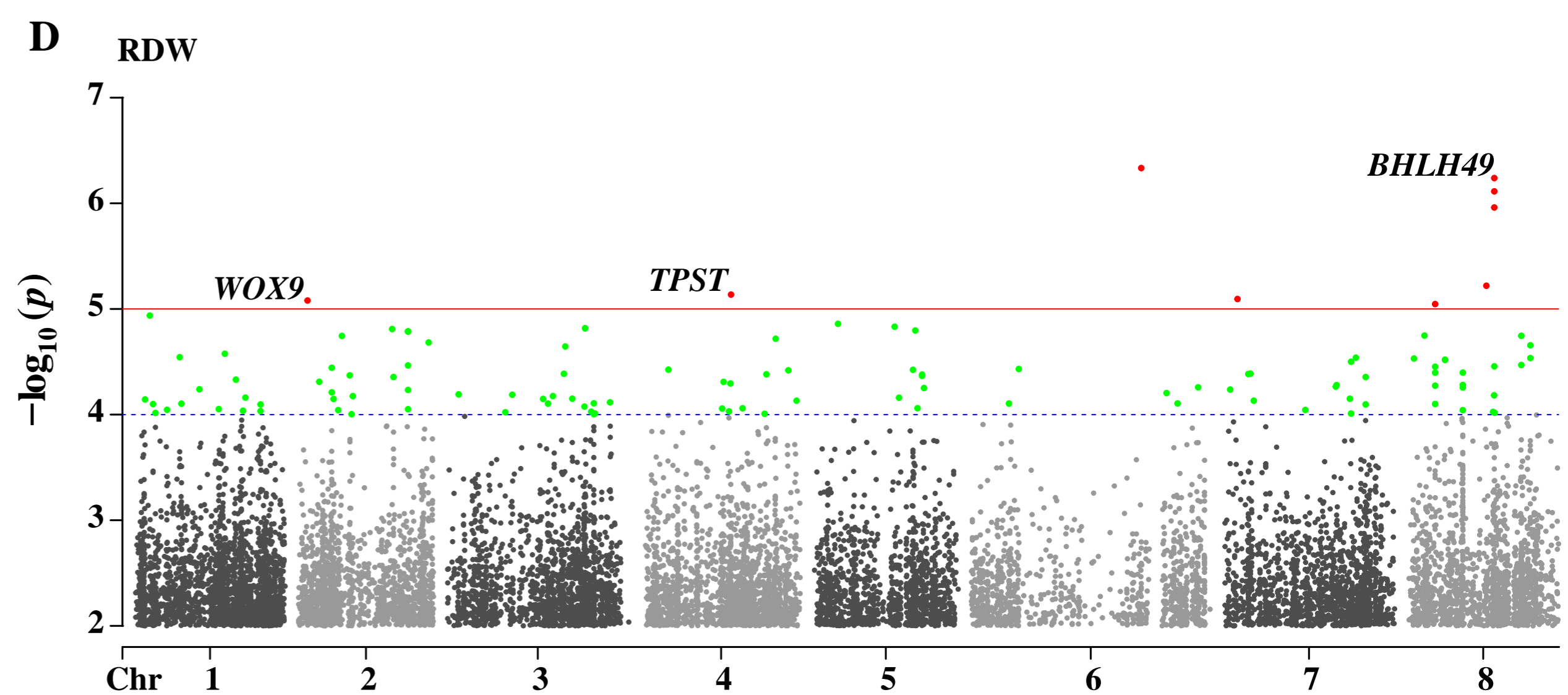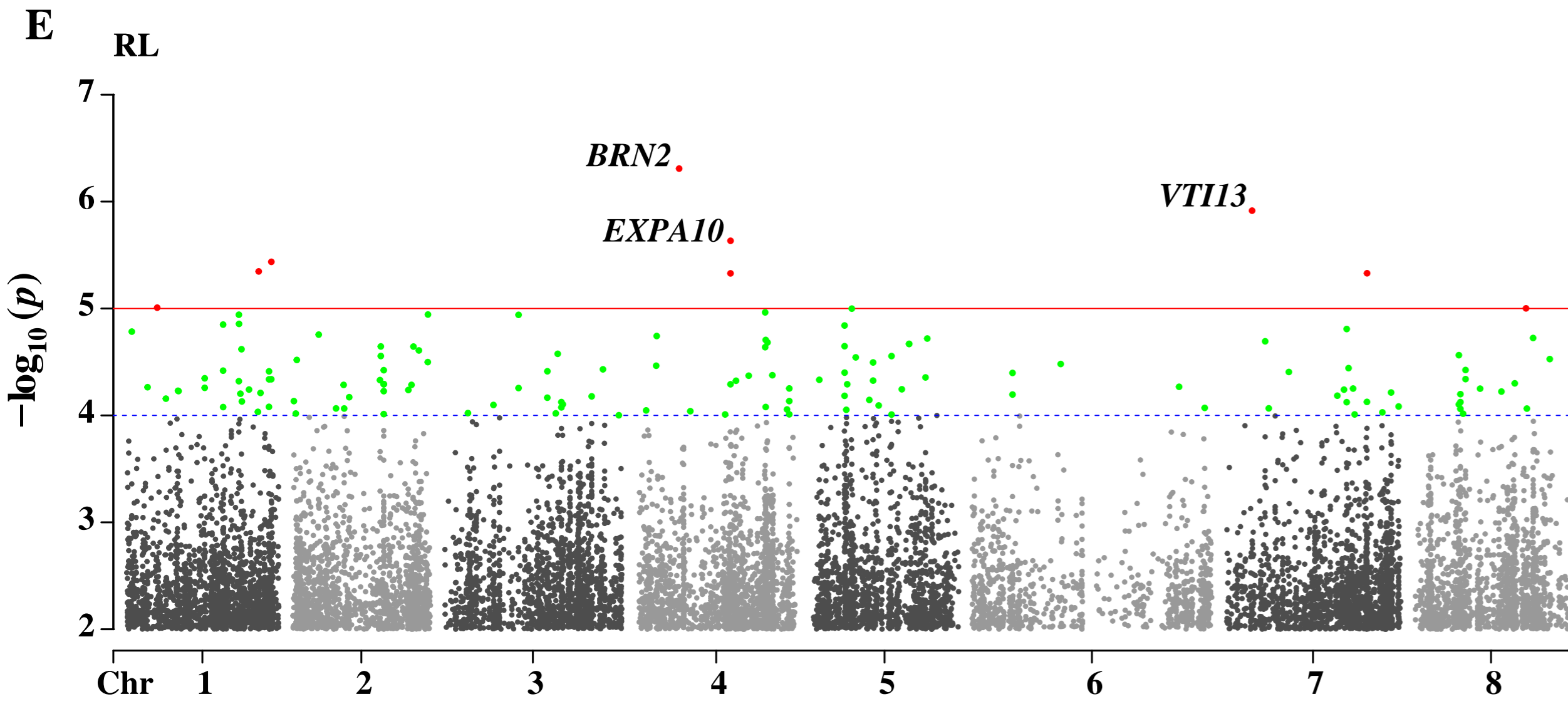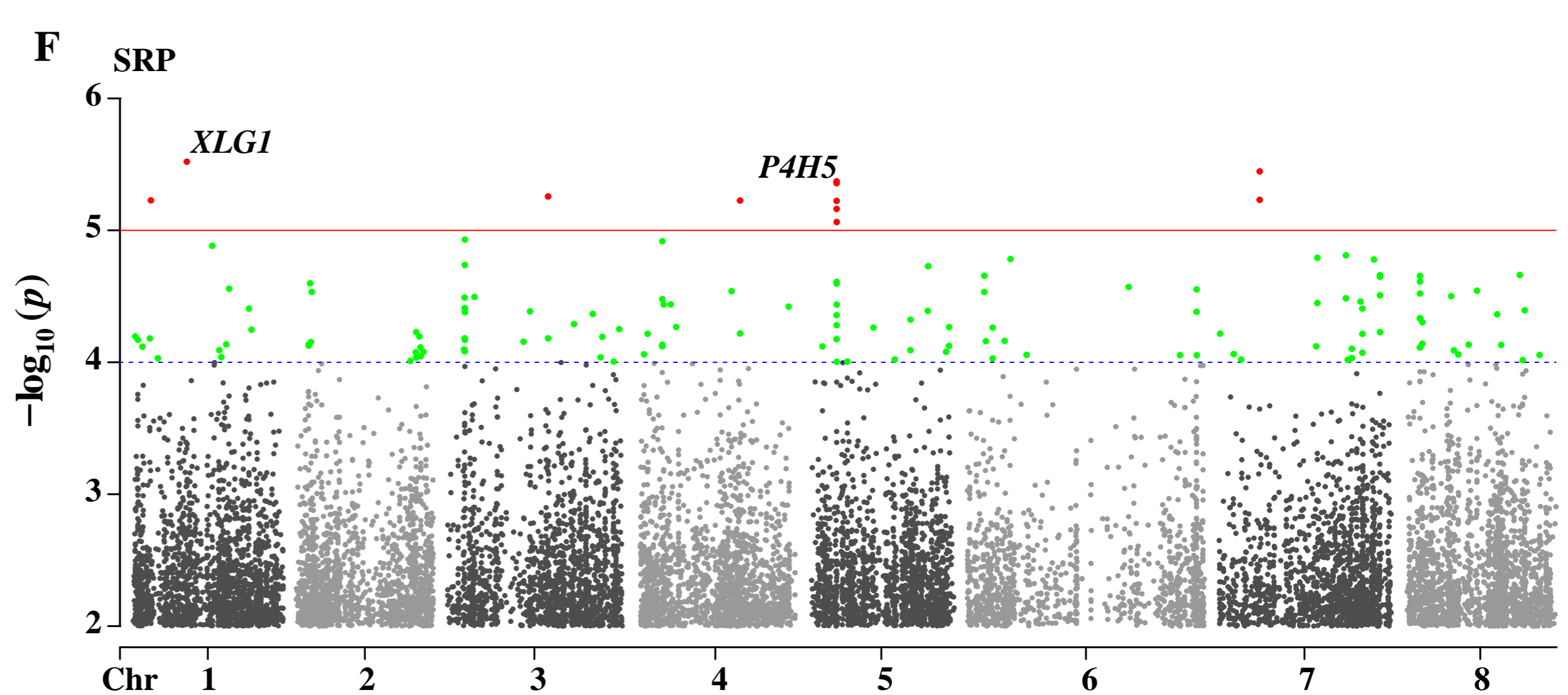

Supplement: Web_Material_uhae271 [file web_material_uhae271.zip › Fig S3.pdf]

**RN** N = 130

mean = 0.25

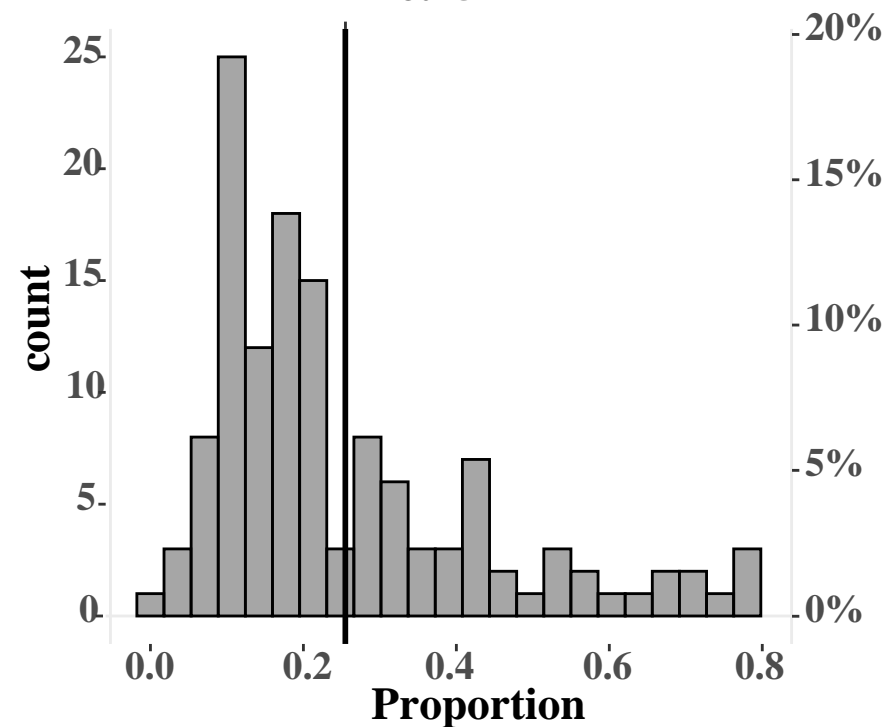

**TRD** N = 109

mean = 0.40

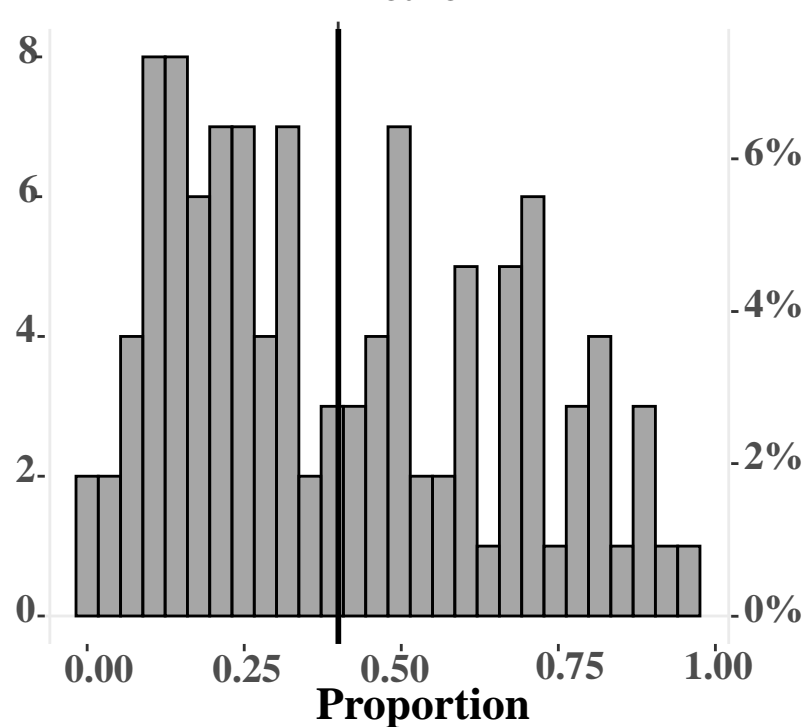

**SRD**

N = 116

mean = 0.49

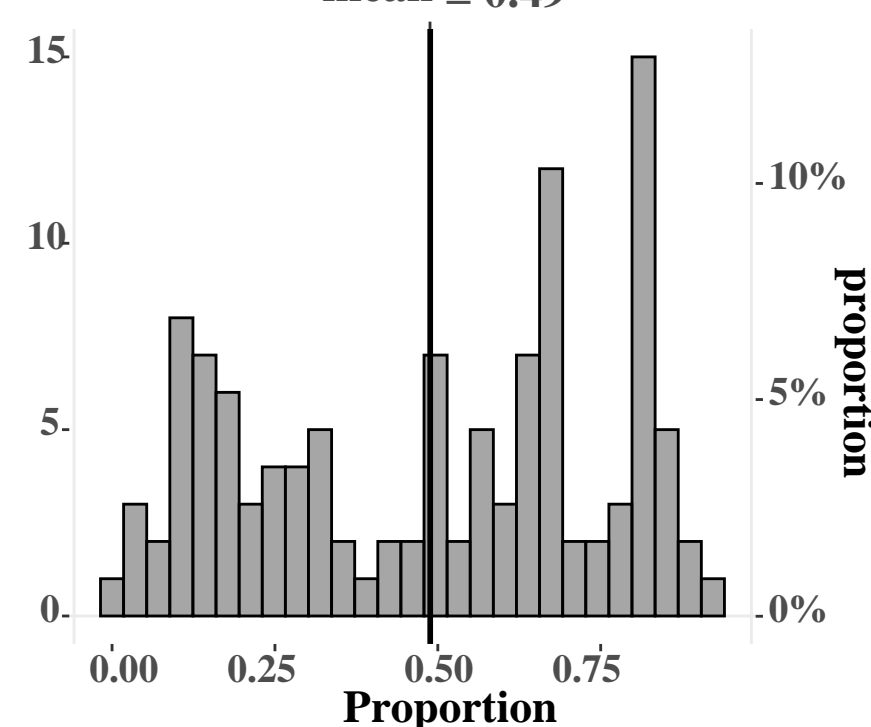

**RDW** N = 111

mean = 0.37

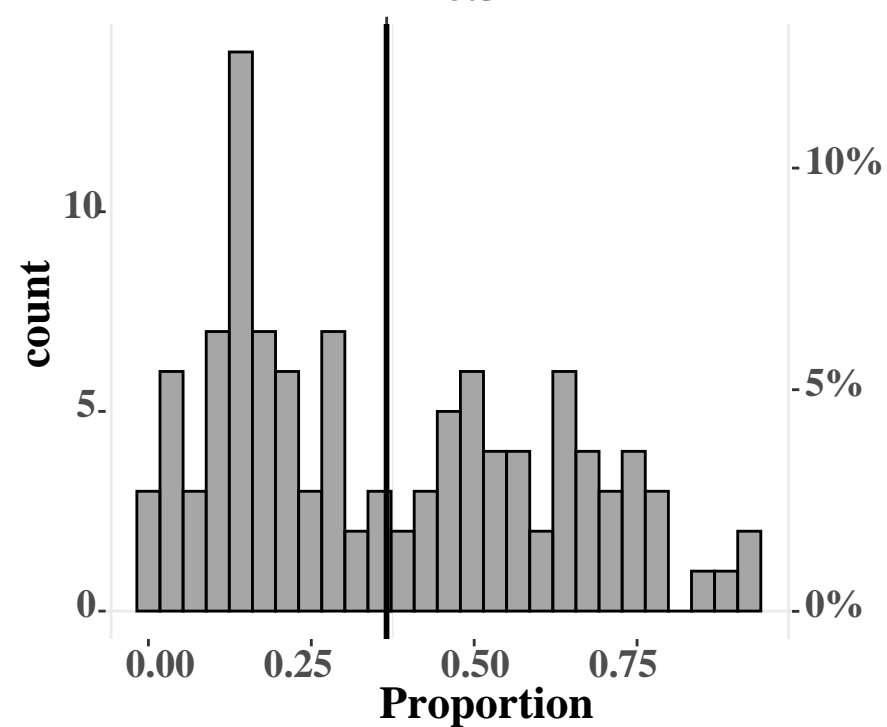

**RL**

N = 140

mean = 0.61

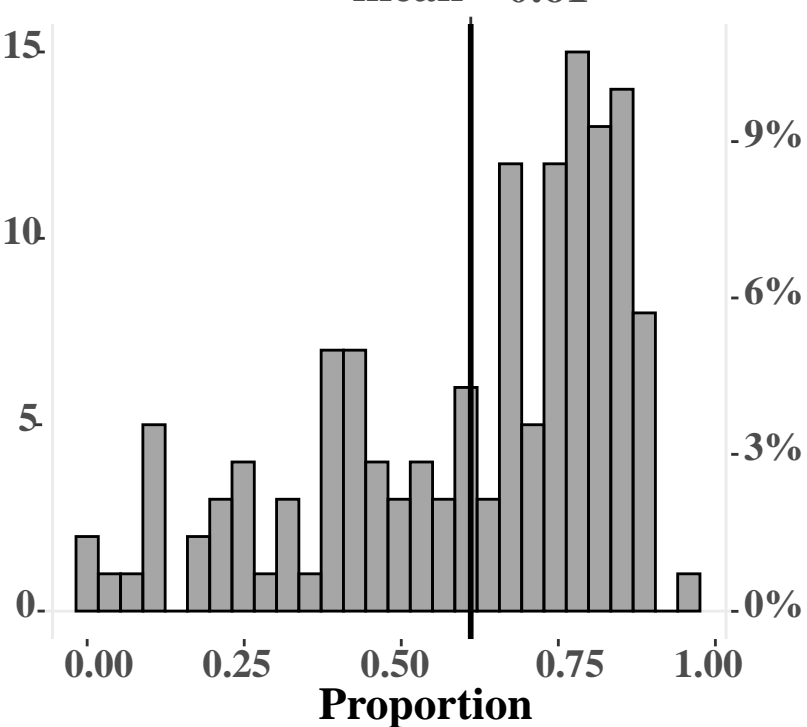

**SRP**

N = 143

mean = 0.42

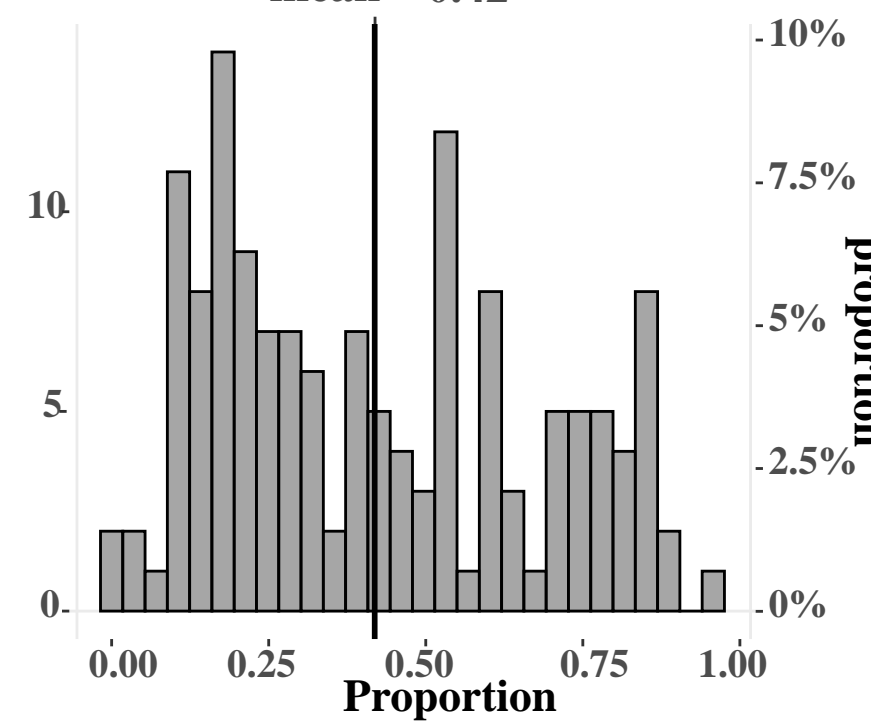

Supplement: Web_Material_uhae271 [file web_material_uhae271.zip › Fig S4.pdf]

**RN**    **N = 130**

**mean = 0.25**

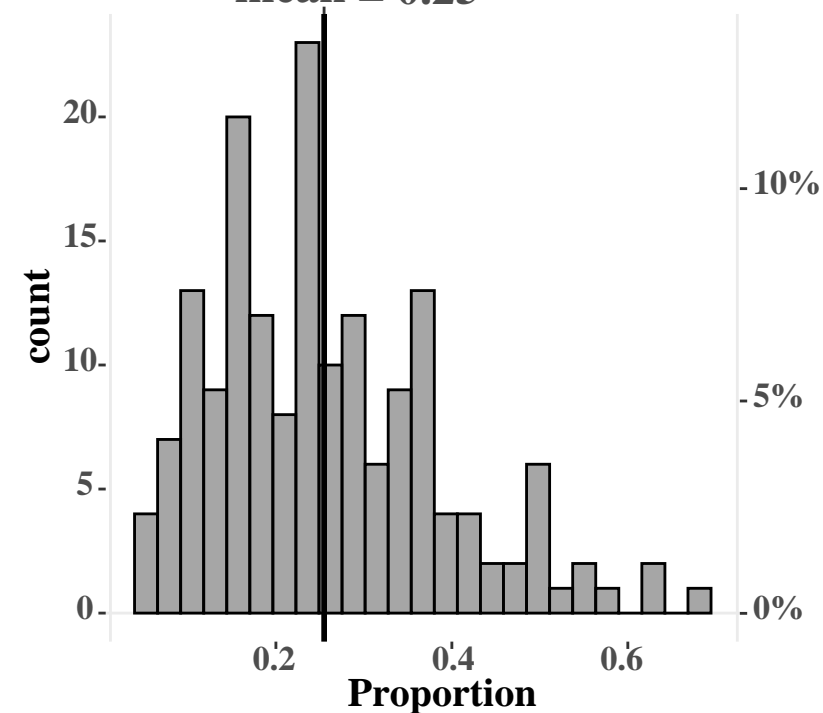

**TRD**    **N = 109**

**mean = 0.40**

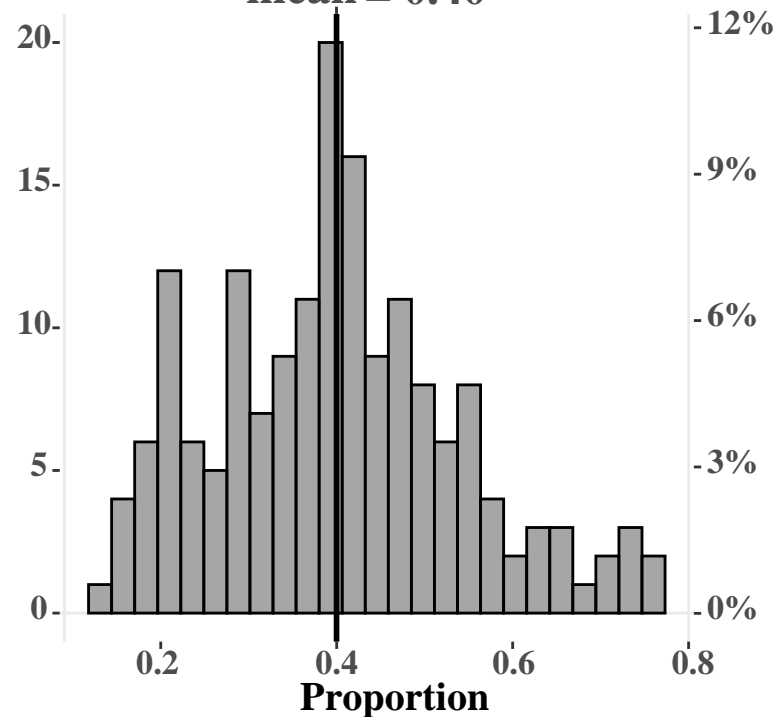

**SRD**

**N = 116**

**mean = 0.49**

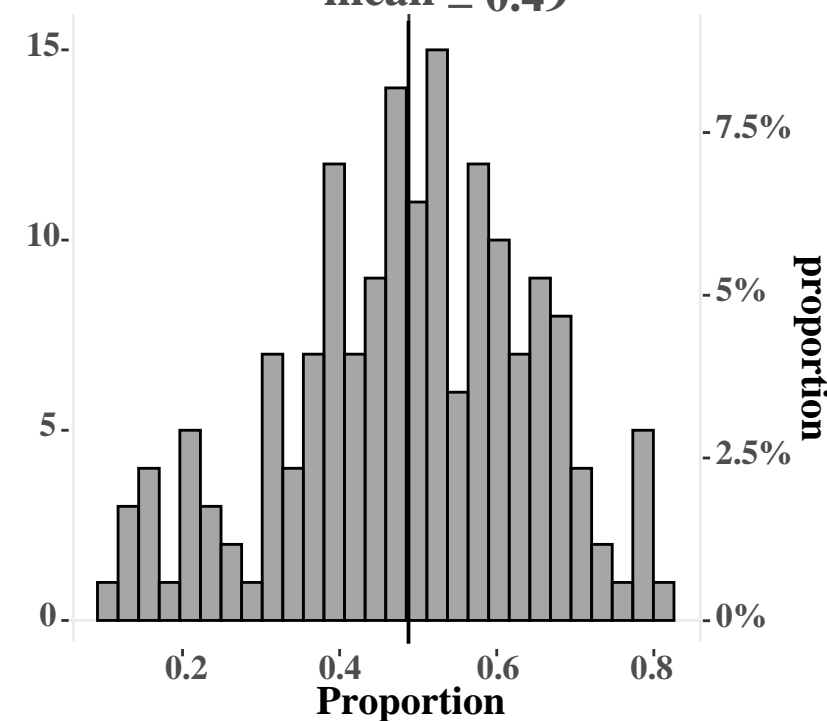

**RDW**    **N = 111**

**mean = 0.37**

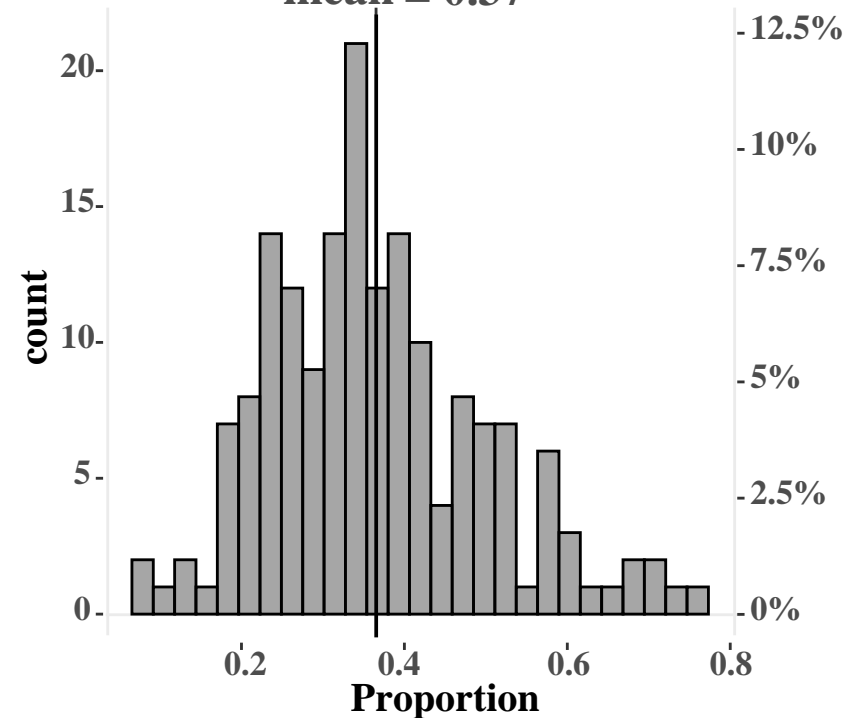

**RL**

**N = 140**

**mean = 0.61**

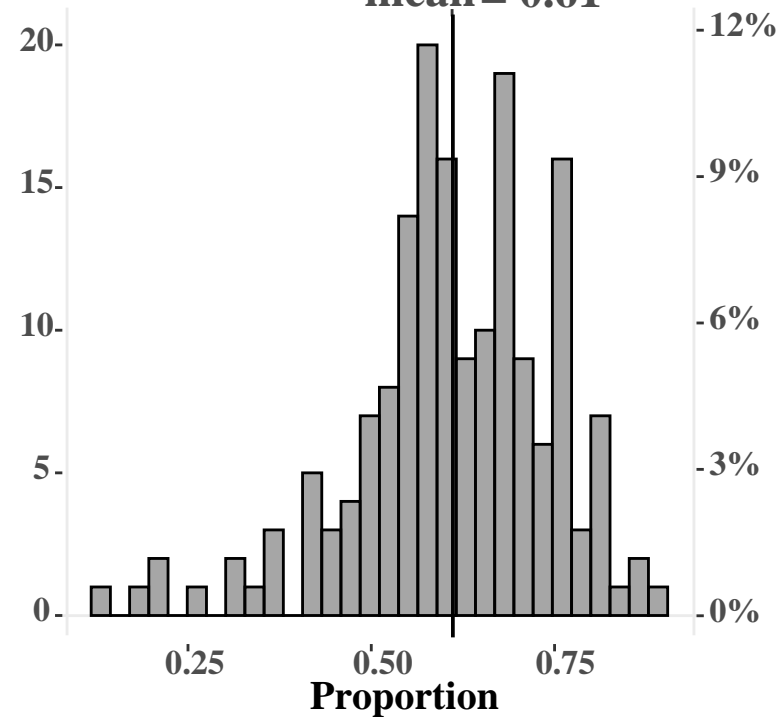

**SRP**

**N = 143**

**mean = 0.42**

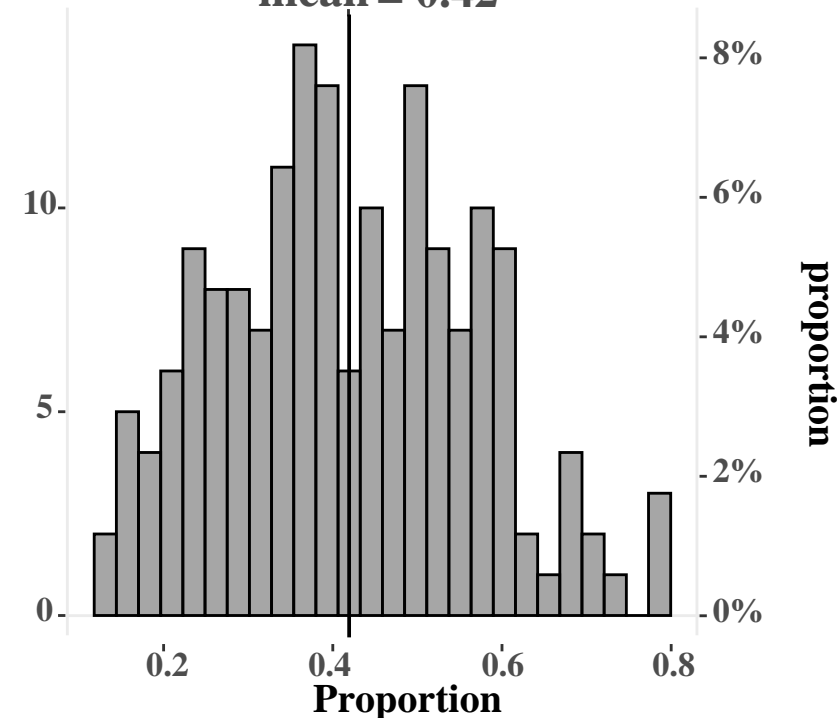

Supplement: Web_Material_uhae271 [file web_material_uhae271.zip › Fig S5.pdf]

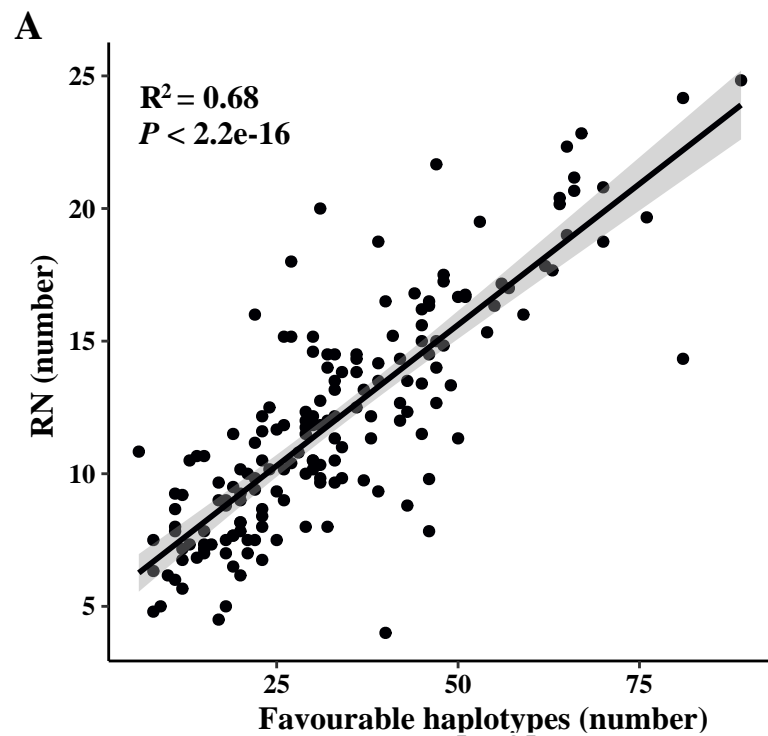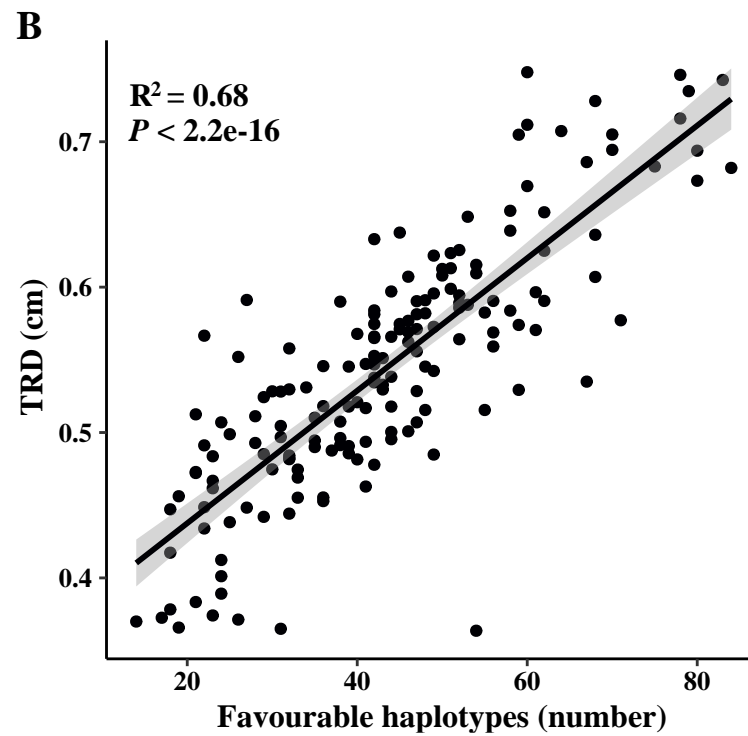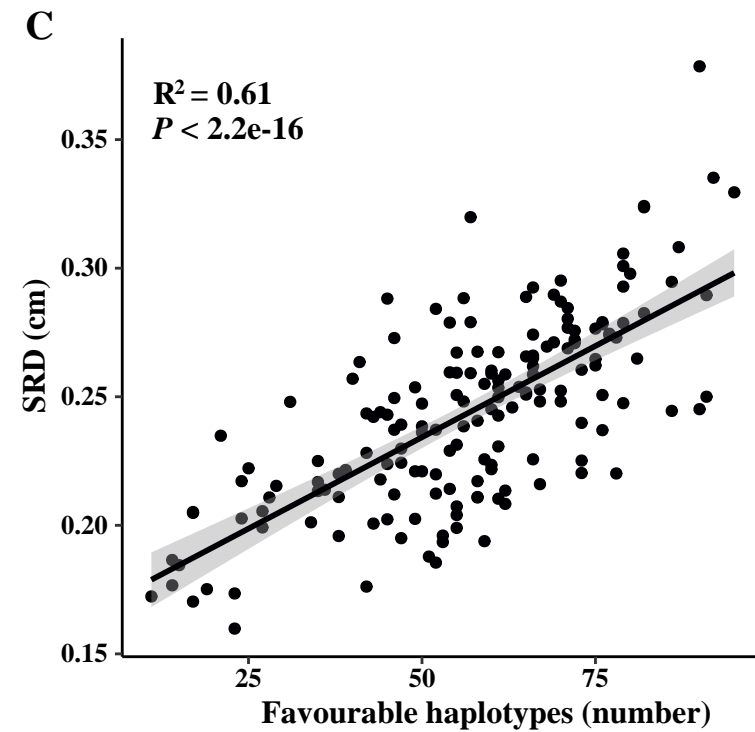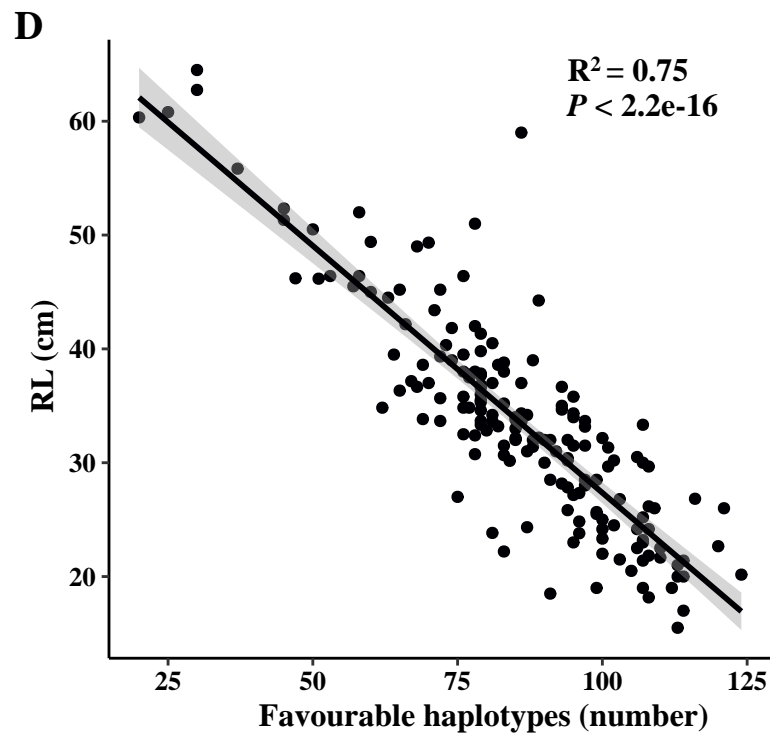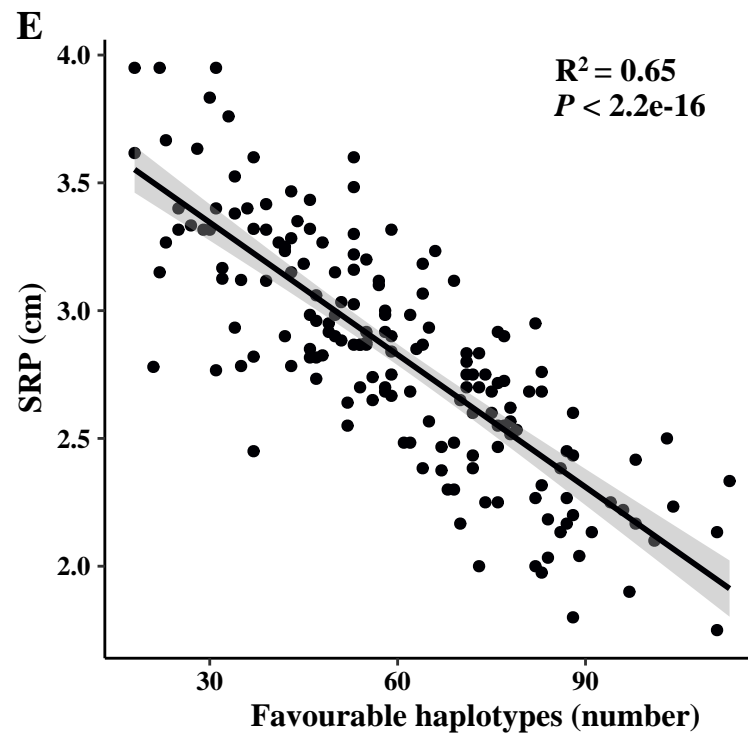

Supplement: Web_Material_uhae271 [file web_material_uhae271.zip › Fig S6.pdf]

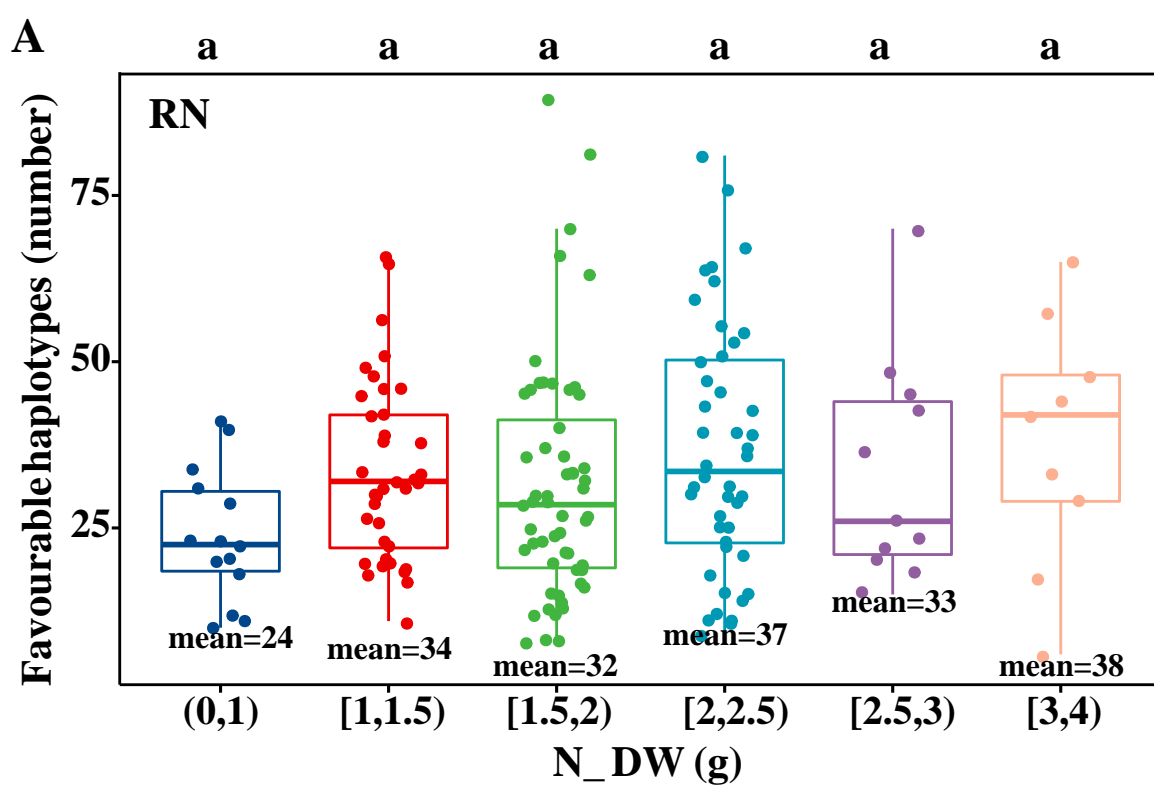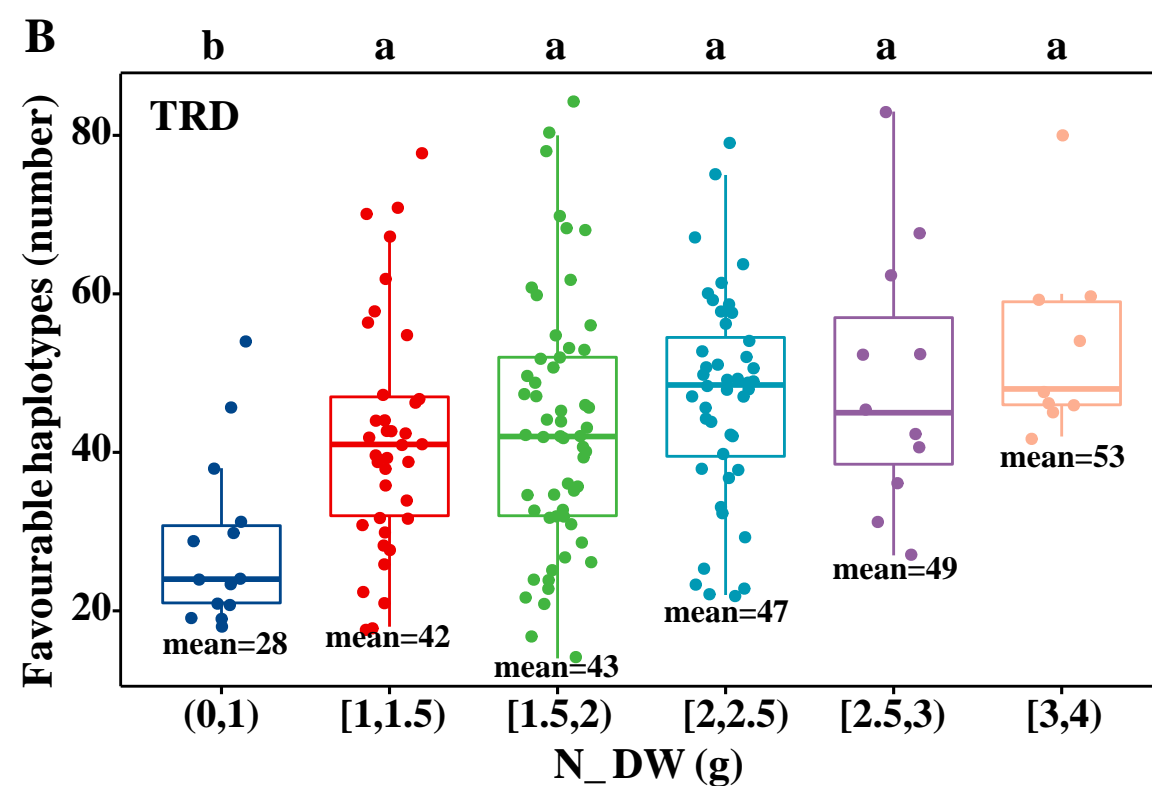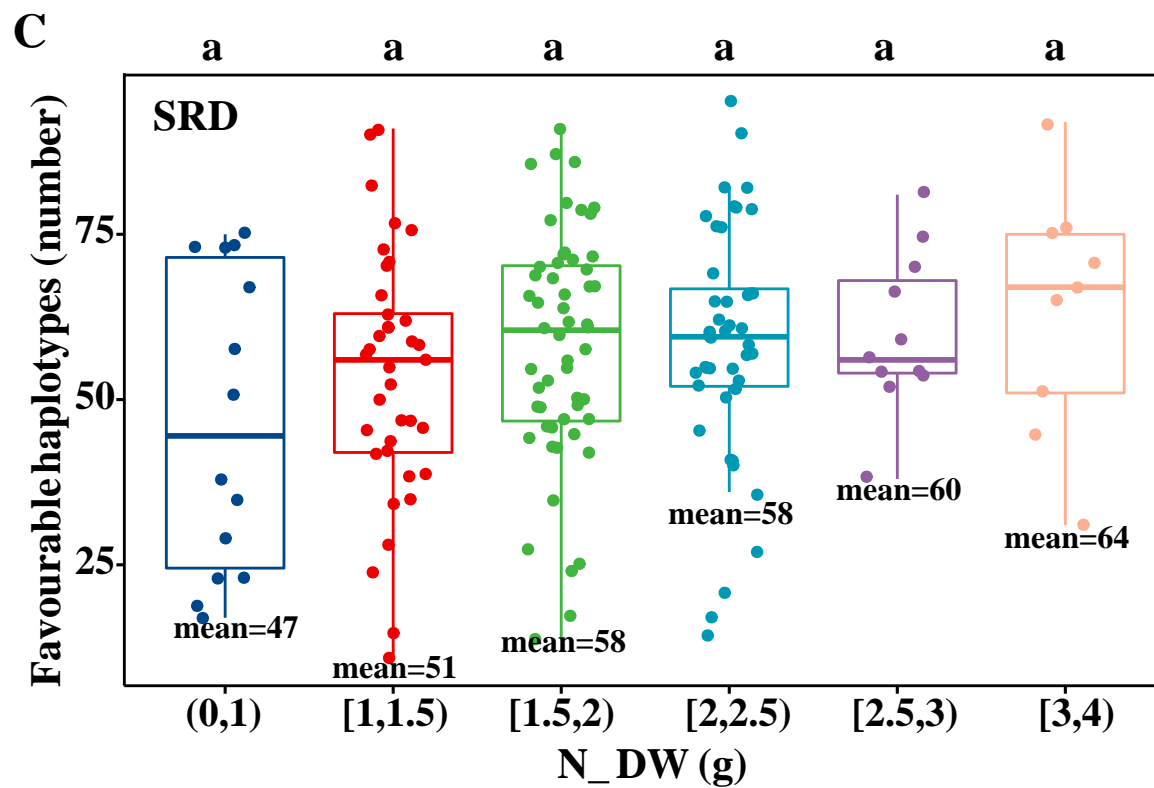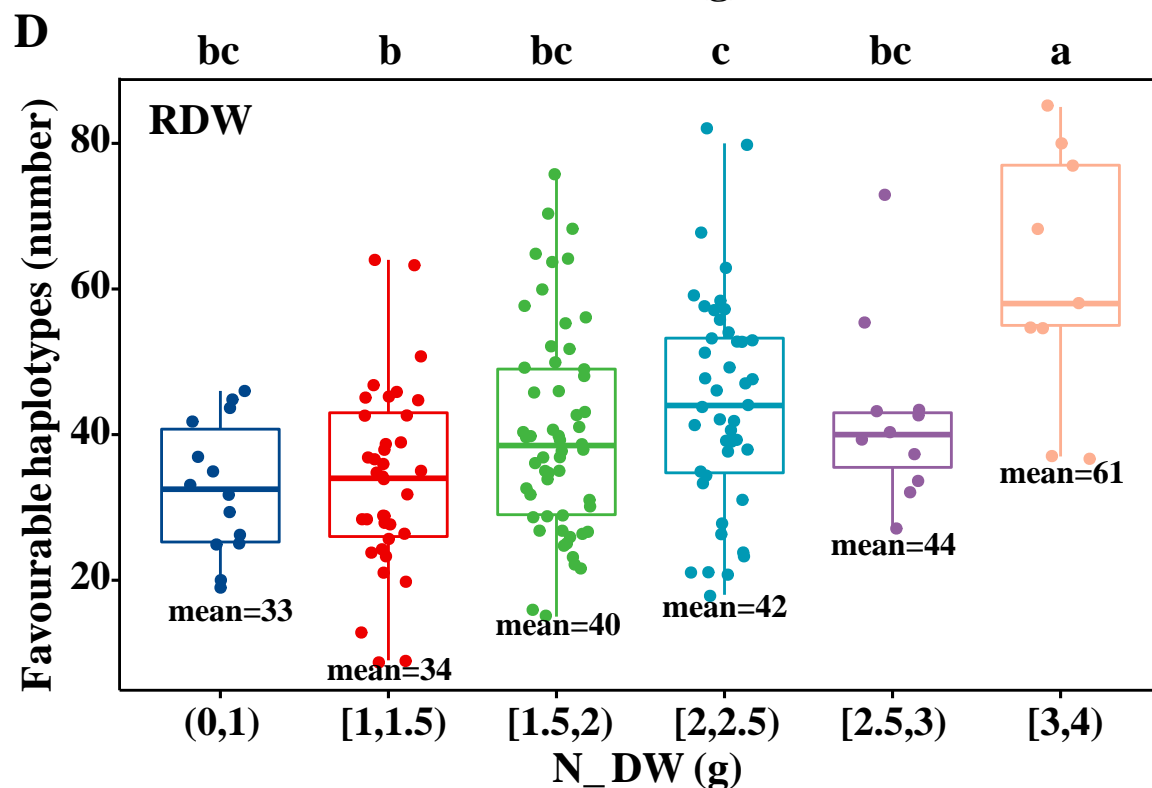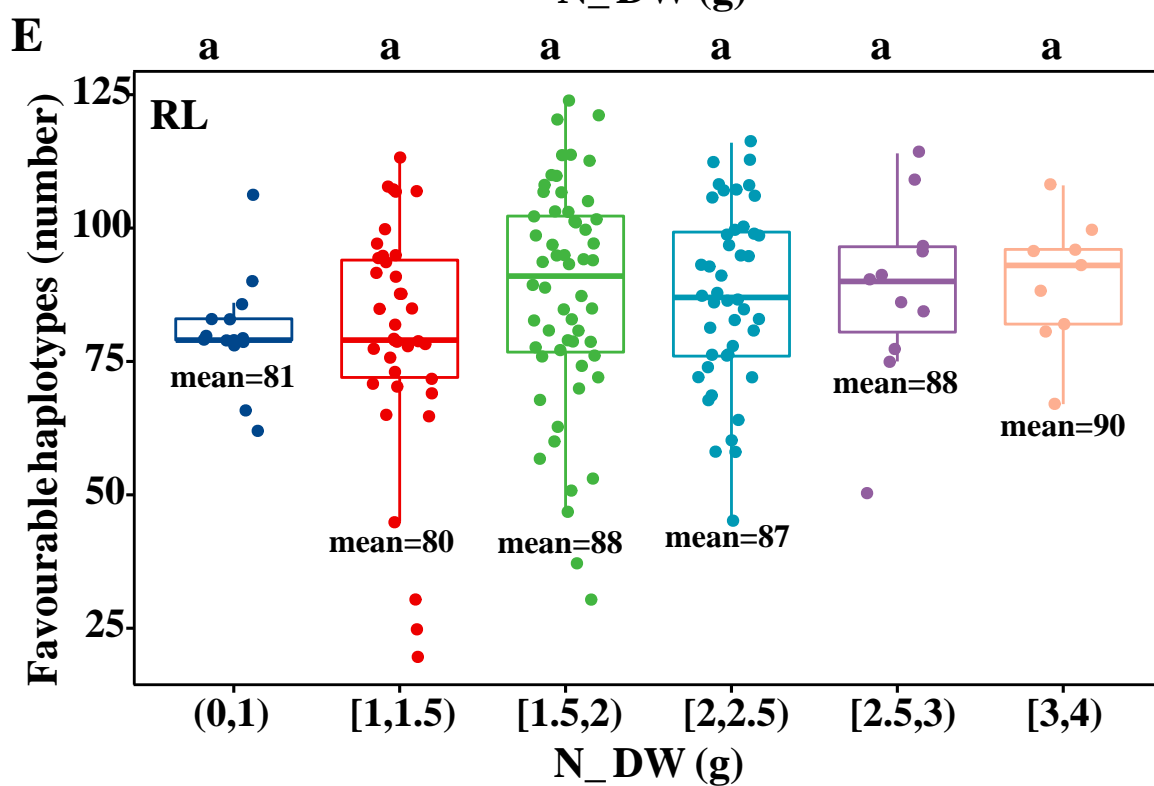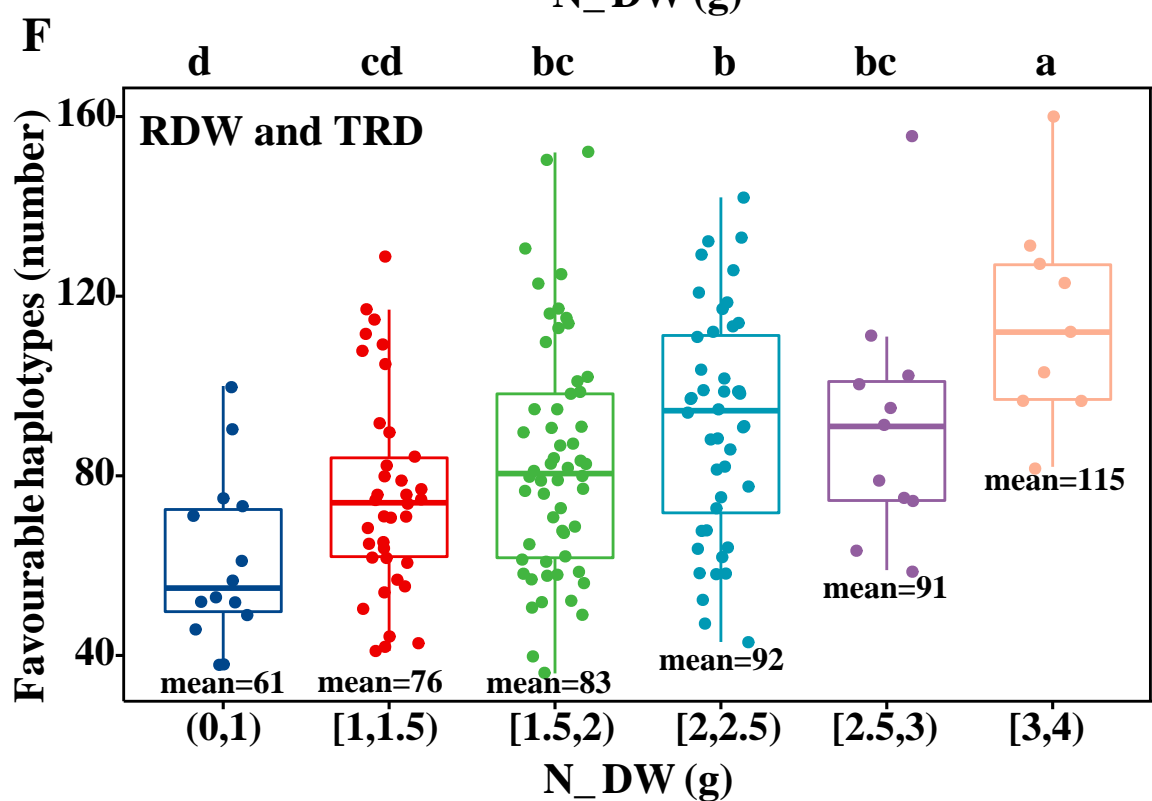

Supplement: Web_Material_uhae271 [file web_material_uhae271.zip › Fig S7.pdf]

**A**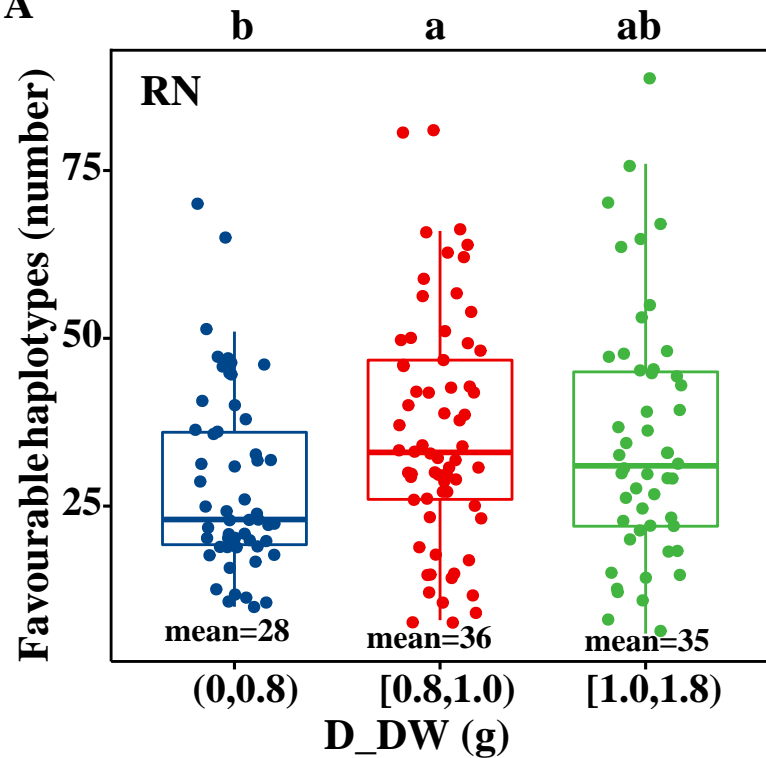**B**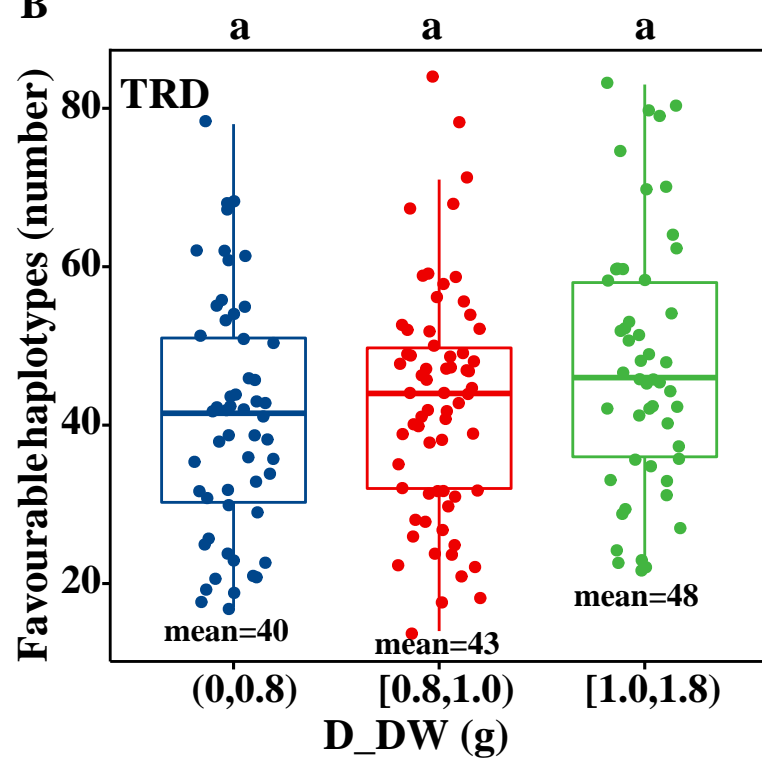**C**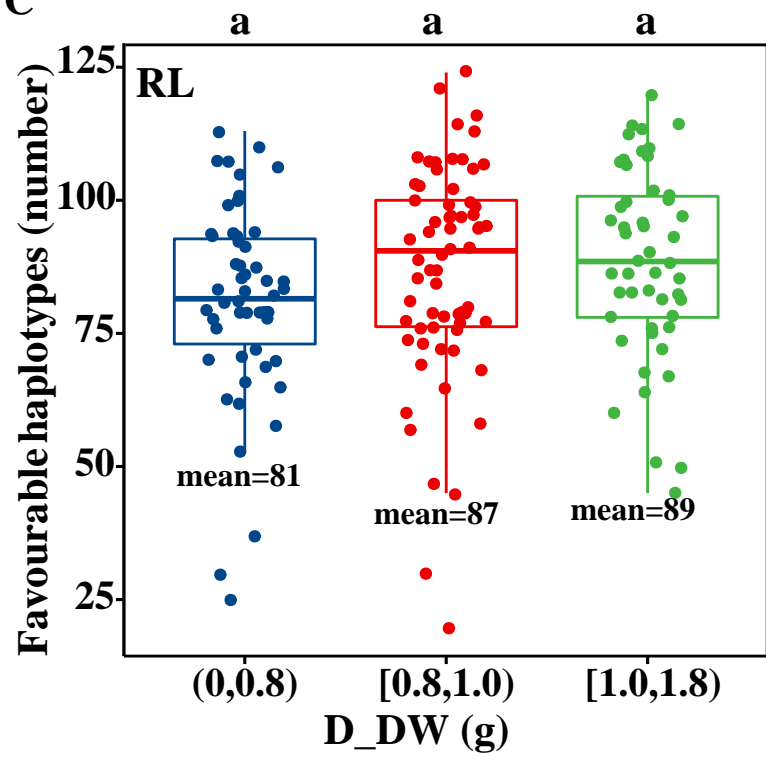

Supplement: Web_Material_uhae271 [file web_material_uhae271.zip › Fig S8.pdf]
